# Global population structure and genotyping framework for genomic surveillance of the major dysentery pathogen, *Shigella sonnei*

**DOI:** 10.1038/s41467-021-22700-4

**Published:** 2021-05-11

**Authors:** Jane Hawkey, Kalani Paranagama, Kate S. Baker, Rebecca J. Bengtsson, François-Xavier Weill, Nicholas R. Thomson, Stephen Baker, Louise Cerdeira, Zamin Iqbal, Martin Hunt, Danielle J. Ingle, Timothy J. Dallman, Claire Jenkins, Deborah A. Williamson, Kathryn E. Holt

**Affiliations:** 1 Department of Infectious Diseases, Central Clinical School, Monash University, Melbourne, VIC Australia; 2 Department of Clinical Infection, Microbiology, and Immunology, Institute for Infection, Ecological and Veterinary Sciences, University of Liverpool, Liverpool, UK; 3 Institut Pasteur, Unité des bactéries pathogènes entériques, Paris, France; 4 Wellcome Sanger Institute, Wellcome Genome Campus, Hinxton, UK; 5 Dept Infection Biology, London School of Hygiene & Tropical Medicine, London, UK; 6 University of Cambridge School of Clinical Medicine, Cambridge Biomedical Campus, Cambridge, UK; 7 Department of Medicine, University of Cambridge School of Clinical Medicine, Cambridge Biomedical Campus, Cambridge, UK; 8 European Molecular Biology Laboratory—European Bioinformatics Institute, Hinxton, UK; 9 Nuffield Department of Medicine, University of Oxford, Oxford, UK; 10 Microbiological Diagnostic Unit Public Health Laboratory, Department of Microbiology and Immunology at the Peter Doherty Institute for Infection and Immunity, The University of Melbourne, Melbourne, VIC Australia; 11 Research School of Population Health, Australian National University, Canberra, ACT Australia; 12 National Infection Service, Public Health England, London, UK; 13 Department of Microbiology, Royal Melbourne Hospital, Melbourne, VIC Australia

**Keywords:** Classification and taxonomy, Phylogenetics, Bacterial genetics, Epidemiology

## Abstract

*Shigella sonnei* is the most common agent of shigellosis in high-income countries, and causes a significant disease burden in low- and middle-income countries. Antimicrobial resistance is increasingly common in all settings. Whole genome sequencing (WGS) is increasingly utilised for *S. sonnei* outbreak investigation and surveillance, but comparison of data between studies and labs is challenging. Here, we present a genomic framework and genotyping scheme for *S. sonnei* to efficiently identify genotype and resistance determinants from WGS data. The scheme is implemented in the software package Mykrobe and tested on thousands of genomes. Applying this approach to analyse >4,000 *S. sonnei* isolates sequenced in public health labs in three countries identified several common genotypes associated with increased rates of ciprofloxacin resistance and azithromycin resistance, confirming intercontinental spread of highly-resistant *S. sonnei* clones and demonstrating the genomic framework can facilitate monitoring the spread of resistant clones, including those that have recently emerged, at local and global scales.

## Abstract (web summary)

Whole genome sequencing is increasingly being adopted for Shigella sonnei outbreak investigation and surveillance, but there is no global classification standard. Here, the authors develop and validate a genomic framework implemented using open-source software, and demonstrate its application using surveillance data.

## Introduction

*Shigella* spp are Gram-negative bacterial pathogens that cause shigellosis (bacterial dysentery). *Shigella* are transmitted via the faecal-oral route and estimated to cause ~188 million infections annually, leading to ~160,000 deaths mainly in young children^1^. In low- and middle-income settings, most of the *Shigella* disease burden of shigellosis is in children under five years^2^, however in high-income countries *Shigella* is frequently detected in returned travellers or men who have sex with men (MSM)^3,4^. *Shigella sonnei* is the most frequently isolated agent of shigellosis in high-income countries and in those that are economically developing^1,5,6^. *S. sonnei* emerged recently (~350 years ago^7^), share a single serotype, and display limited genomic diversity (all belong to ST152 complex by multi-locus sequence typing (MLST)). These properties make it difficult to differentiate and track *S. sonnei* strains^7^, motivating adoption of whole-genome sequencing (WGS) for research and public health surveillance of this organism^8^. Core-genome MLST (cgMLST) is available via the *Escherichia coli* scheme in EnteroBase^9^ but has not been widely adopted for *S. sonnei* surveillance, and most public health labs and research studies rely on the higher-resolution technique of single nucleotide variant (SNV)-based phylogenetics analysis.

The global population of *S. sonnei* is divided into five major lineages^7,10^. Several WGS studies have investigated regional *S. sonnei* epidemiology and population structure, including in Asia^11–14^, Australia^4^, the Middle East^15^, South America^10^, and the United Kingdom^16–18^; and defined additional sub-lineage-level phylogenetic groups of local epidemiological importance, associated with features such as ciprofloxacin-resistance^19^, transmission within Orthodox Jewish communities^15^, or transmission amongst MSM^4,17^. *S. sonnei* from Asia, Europe, Australia and North America have for >20 years been dominated by Lineage 3 strains that are resistant to early first-line antimicrobials (trimethoprim-sulfamethoxazole, tetracycline, and streptomycin) due to the presence of antimicrobial resistance (AMR) genes acquired horizontally via the small plasmid spA and a chromosomal Tn*7*-like transposon^7,20,21^. Resistance to chloramphenicol and/or ampicillin is also observed (e.g., via acquisition of the *Shigella* resistance locus (SRL)^10,22^). Reduced susceptibility to fluoroquinolones has emerged on multiple occasions and in multiple locations through acquisition of point mutations within the quinolone resistance determining region (QRDR) of *gyrA*^7,11^. Resistance to ciprofloxacin has emerged at least once via the accumulation of three QRDR mutations (2 in *gyrA* and one in *parC*) in a South Asian sublineage that has since been detected on multiple continents^12–14,23^. Resistance to the last few remaining drugs is increasing through the acquisition and maintenance of plasmids carrying *mph(A)* and *ermB* (azithromycin resistance) or extended-spectrum beta-lactamase (ESBL) genes (ceftriaxone resistance), often in combination with additional aminoglycoside resistance genes^11,14,16,18,24^.

In many countries, *S. sonnei* is a notifiable infection and subject to public health surveillance and outbreak investigations, which are increasingly conducted using WGS^8,25–28^. However, the lack of a defined global genomic framework and accompanying genotype nomenclature hampers both local reporting, outbreak detection, and patterns of spread within regions. For example, most *S. sonnei* WGS studies have reported which of the five major lineages their novel isolates belong to, but have had to download public reference genome data, construct whole genome alignments, and infer phylogenies to achieve this basic identification^23,27^. Studies of MSM *S. sonnei* in different settings have designated different names for the same lineages^4,16,18^, obscuring the fact that the same clones are spreading amongst MSM communities in different countries, and the only way to recognize this currently is through construction of whole-genome phylogenies incorporating data from multiple prior studies^29^.

WGS-based genotyping frameworks based on single nucleotide variants (SNVs) have been widely adopted for the bacterial pathogens *Mycobacterium tuberculosis*^30^ and *Salmonella enterica* serovar Typhi^31^, which display similarly low levels of genomic diversity to *S. sonnei*. These frameworks enable fast and accurate typing of clinical isolates from WGS data without the need for time-consuming comparative genomics or phylogenetics, facilitating straightforward identification of (and cross-jurisdictional communication about) epidemiologically important lineages from WGS data.

Here, we describe the global population structure for *S. sonnei* and (i) propose a hierarchical SNV-based genotyping scheme, which we define using 1935 globally distributed genomes; (ii) implement the scheme within the free and open-source Mykrobe^32^ software alongside detection of genetic determinants that are highly predictive of fluoroquinolone susceptibility phenotypes in *S. sonnei*^28^; and (iii) validate this approach to genotyping using an additional 2015 genomes that were sequenced in public health laboratories and deposited in the publicly available GenomeTrakr database. Code is available at https://github.com/katholt/sonneityping. By applying this novel genotyping framework to *S. sonnei* WGS data generated in public health laboratories on three continents, we demonstrate the utility of the new scheme for tracking and reporting emerging AMR clones both within and between jurisdictions.

## Results

### Defining phylogenetically informative genotypes for *S. sonnei*

**Fig. 1:**
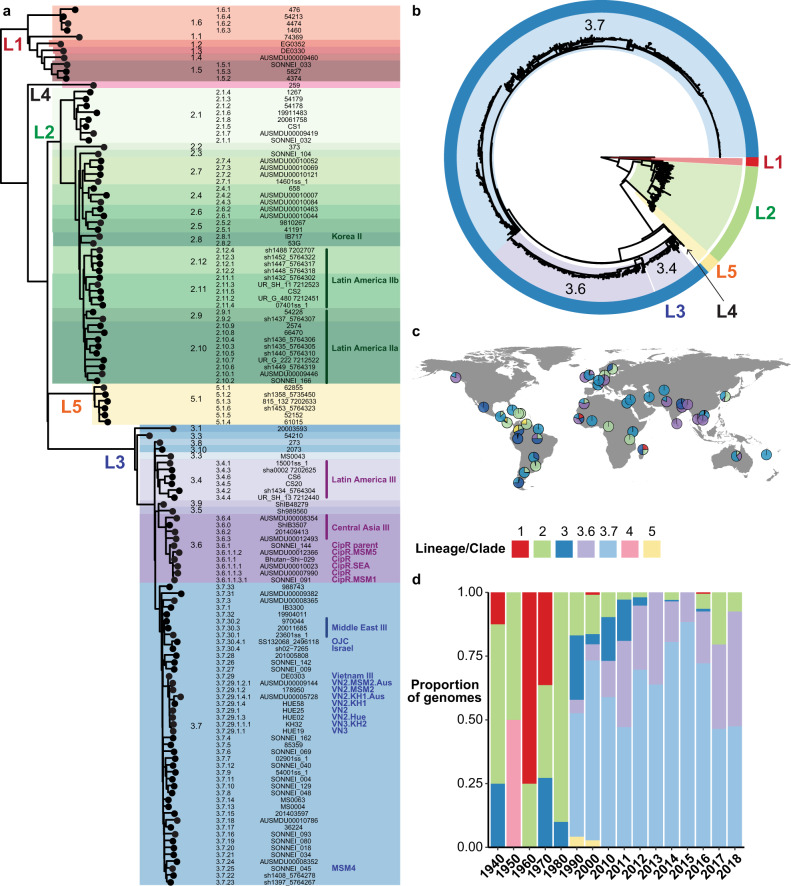
Population structure, temporal distribution and geographic distribution of the 1935 *S. sonnei* genomes in the discovery set. **a** Maximum likelihood phylogeny (outgroup rooted using *E. coli*) of one representative per genotype. Lineages are labelled L*X*, where *X* is the lineage number. Highlighting and column 1 indicate clades, column 2 indicates genotype, column 3 shows strain names, column 4 shows human readable genotype names (for epidemiological groups noted in Table 2). **b** Maximum likelihood phylogeny (outgroup rooted using *E. coli*), **c** frequencies by geographic region, and **d** frequencies by decade/year; for all discovery set genomes and coloured by lineage and major clades (3.6, 3.7, see inset legend). Interactive version of linked phylogeny, map and timeline for this data set are available online in Microreact (https://microreact.org/project/fG2N7huk9oZNCaVHu8rukr).

In order to define the global population structure and identify clades and marker SNVs, we collated 1935 publicly available *S. sonnei* genomes from eight studies^4,7,10–13,15,16^ as our “discovery” dataset (see Supplementary Table 1). These genomes represent isolates from 48 countries, collected between 1943 and 2018 (Fig. 1c, d, Table 1). The majority originate from Asia (32.4%), Europe (29.3%), Australia (18.8%), or Latin America and the Caribbean (17.4%) (Table 1). The data set is diverse in terms of acquired AMR genes (median 9 per genome, range 0–21), and includes 150 (7.8%) genomes known to be associated with MSM.

**Table 1:** Geotemporal distribution of 1935 *S. sonnei* genomes in the discovery dataset.

	Region	Country	Year(s) of isolation	No. of isolates
Africa	Total		1967–2006	19 (0.98%)
	Central Africa	Cameroon	1973	1
	East Africa	Madagascar	1998–2000	3
		Kenya, Tanzania	2004	2
	Northern Africa	Egypt	2005–2006	4
		Morocco	2005–2006	4
	West Africa	Senegal	1967–2006	4
		Burkina Faso	2006	1
Asia	Total		1979–2014	627 (32.4%)
	Central Asia	Uzbekistan	2005	1
	Eastern Asia	Korea	1979–2003	20
	Southern Asia	Bhutan	2011–2013	71
		India	2013–2014	24
		Pakistan	2002–2003	7
		Other (Nepal, Iran, Sri Lanka)	2003–2006	3
	Southeast Asia	Cambodia	2013–2014	4
		Thailand	1994–2013	9
		Vietnam	1995–2015	266
	Western Asia	Israel	1992–2014	222
Europe	Total		1943–2016	567 (29.3%)
	Northern Europe	United Kingdom	1990–2016	393
		Ireland	Unknown	5
		Sweden	1943–1947	6
		Denmark	1945	1
	Western Europe	France	1945–2014	158
		Belgium	2008	3
		Germany	Unknown	1
Northern America	Total		1994–2015	16 (0.8%)
	North America	USA	2004–2015	13
		Other (unknown)	1994–1995	3
Latin America and the Caribbean	Total		1997–2014	337 (17.4%)
	Caribbean	Cuba, Dominican Republic, Haiti	2003–2006	3
	Central America	Costa Rica	2002–2010	50
		Guatemala	2011–2012	30
		Mexico	1998	1
	South America	Argentina	2002–2011	50
		Brazil	1997–2002	7
		Chile	2010–2011	27
		Colombia	2008–2011	30
		French Guiana	1998–2006	3
		Paraguay	2008–2012	18
		Peru	1999–2012	49
		Uruguay	2000–2011	28
		Venezuela	1997–2014	41
Australia and Oceania	Total		1997–2018	364 (18.8%)
	Australia	Australia	2016–2018	363
	Oceania	New Caledonia	1997	1
Unknown	Total		Unspecified	5 (0.5%)

**Fig. 2:**
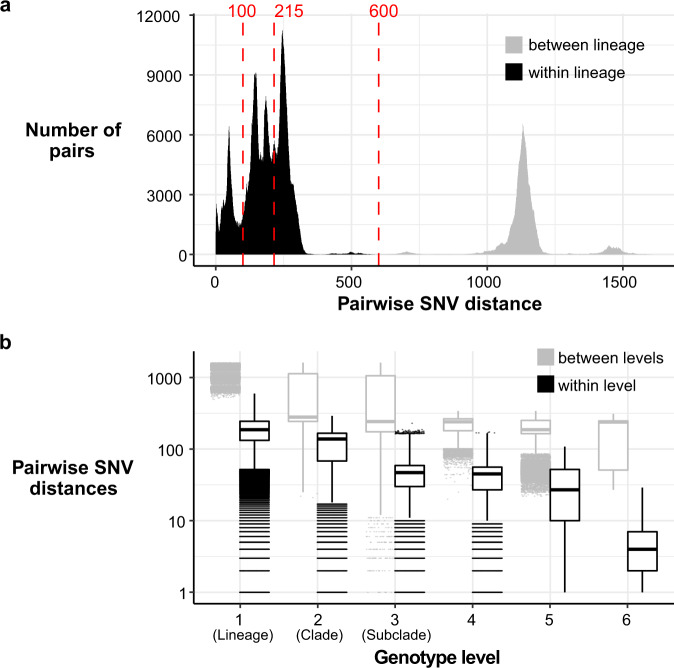
Distribution of SNV distances in discovery genomes. **a** Histogram of pairwise SNV distances between all discovery genomes, coloured by lineage comparison as per legend. Red lines mark SNV cut-offs used to define lineage, clade and subclade levels in genotyping scheme. **b** Boxplots of pairwise SNV distances (log scale, *n* = 1,873,081 pairwise distances) between discovery genomes at different hierarchical levels of the defined genotyping scheme. Boxes indicate the median (bold line), 25th to 75th percentiles (box), and the 5th and 95th percentile (whiskers), with outliers shown as points. Lineage, Clade and Subclade refer to the first three levels of the scheme. ‘4’ indicates the fourth level of the scheme (i.e., the final ‘1’ in 3.6.1.1), ‘5’ the fifth level (i.e., the ‘1’ in 3.7.30.4.1), and ‘6’ the sixth level (i.e., the final ‘1’ in 3.6.1.1.3.1).

The recombination-filtered core-genome maximum likelihood phylogeny inferred from these genomes (Fig. 1b) was robust (median bootstrap support 100%) and exhibited the five previously-described deep branching lineages^7,10^. Lineage 3 was most common (86.9%), followed by Lineage 2 (10.7%), Lineage 5 (1.4%), Lineage 1 (0.9%) and Lineage 4 (*n* = 1). The pairwise core-genome SNV distance distribution revealed peaks and troughs which we used to set thresholds to define clusters at different levels of resolution (Fig. 2a). A threshold of 600 pairwise SNVs separated the five major lineages; troughs at 215 SNVs and 100 SNVs were used to define higher-resolution genetic clusters. (A similar structure was recovered using hierarchical Bayesian clustering of the SNV matrix using FastBAPS, but with less consistent levels of divergence between clusters; see Supplementary Fig. 1). Mapping the pairwise SNV threshold-defined clusters onto the phylogeny confirmed that each cluster corresponded to a monophyletic group with 100% bootstrap support, which we designate as clades (*n* = 29, using 215-SNV threshold) and subclades (*n* = 96, using 100-SNV thresholds).

We used these cluster memberships to define hierarchical genotypes with nomenclature in the form [lineage].[clade].[subclade] (see Fig. 1a). Similar to the *M. tuberculosis* and *S*. Typhi schemes, this hierarchical nomenclature facilitates easy recognition of relationships between genotypes; e.g., subclades 3.6.1, 3.6.2, …, 3.6.N are sister groups in the whole-genome phylogeny, nested within clade 3.6, which falls within Lineage 3 (see Fig. 1a). The median pairwise distance between genomes of the same clade or subclade was 138 or 47 core-genome SNVs, respectively (Fig. 2b).

Whilst the discovery set is not a systematic sampling across geographic regions, it can provide some preliminary insights into the global distribution of *S. sonnei* genotypes. Lineages were broadly distributed across continents (with the exception of Lineage 5 and the singleton Lineage 4, see Fig. 1c, Supplementary Data 1), however the majority of clades (*n* = 24, 83%) were represented by isolates from just one (*n* = 16, 57%) or two (*n* = 7, 23%) continents. At the other extreme, clades 3.6 and 3.7 were widely distributed, with representatives on all six continents (Fig. 1b). Clades 3.6 and 3.7 were the most common overall (19% and 62%, respectively) and accounted for the majority of *S. sonnei* from all continents except Latin America, where clades 2.10, 2.11, 2.12, 3.4, 3.6 and 5.1 were common (8–28% each). Subclades showed even greater geographic specificity, with 74% (*n* = 71) represented by a single continent only and 72% (*n* = 69) represented by a single region only. Fifty-nine subclades (61%) were dominated by genomes from a single country.

As a key goal of the *S. sonnei* genotyping scheme is to facilitate identification and communication about subtypes of public health interest, we reviewed the position of genetic clusters that have been described in the literature as being of epidemiological importance (Table 2). Groups previously identified as being associated with specific geographical regions mapped mainly to clades or subclades defined in the genotyping scheme (Table 2). Most groups previously defined on the basis of AMR or transmission patterns comprised more recently-emerged clusters, forming monophyletic groups within our subclade-level genotypes. Hence we created additional higher-resolution genotypes nested within subclades to demarcate these groups (e.g., 3.6.1.1, 3.6.1.1.1; see Table 2), and anticipate adding more genotypes as new resistant groups emerge in future. For example, the ciprofloxacin resistant triple-mutant sublineage^12–14^ comprised a monophyletic group within subclade 3.6.1 that we define as genotype 3.6.1.1; distinct subgroups within this have also been described, associated with South East Asia (genotype 3.6.1.1.1), and MSM communities in Australia (3.6.1.1.2) or the UK (3.6.1.1.3.1) (see Table 2).

**Table 2:** Details of epidemiological clusters defined within the *S. sonnei* population.

New genotyping framework		Previously defined as		Description
Genotype	Name	Name	Study	
Lineage II				
2.8	Korea II	Korea II	Holt 2012	Associated with Korea
2.9, 2.10, 2.11	Latin America II	South America II LA sublineage IIa & IIb	Holt 2012 Baker 2017	Associated with Latin America
Lineage III				
3.4	Latin America III	South America III LA sublineage IIIa & IIIb	Holt 2012 Baker 2017	Associated with Latin America
3.6	Central Asia III	Central Asia IIIa	Holt 2012	Associated with Central Asia
3.6.1	CipR parent	–	This study	Subclade from which ciprofloxacin-resistant sublineage emerged
3.6.1.1	CipR	Ciprofloxacin-resistant Pop2	The 2015 The 2019	Ciprofloxacin-resistant triple mutation sublineage
3.6.1.1.1	CipR.SEA	-	This study	Ciprofloxacin-resistant isolates associated with South East Asia
3.6.1.1.3.1	CipR.MSM1	MSM clade 1	Baker 2018	MSM-linked ciprofloxacin resistant isolates
3.6.1.1.2	CipR.MSM5	BAPS1 MSM Clade 5	Ingle 2019 Bardsley 2020	MSM-linked ciprofloxacin resistant isolates
3.7.25	MSM4	MSM Clade 4	Baker 2018	MSM-linked
3.7.29	Vietnam III	VN clone	Holt 2012	Associated with South East Asia
3.7.29.1	VN2	VN clone, sweep 2	Holt 2013	Clonal group originating from genetic sweep 2
3.7.29.1.1	VN3	VN clone, sweep 3	Holt 2013	Clonal group originating from genetic sweep 3
3.7.29.1.1.1	VN3.KH2	KH2	Holt 2013	Kanh Hoa subclone 2, emerging from sweep 3
3.7.29.1.1.2	VN4	VN clone, sweep 4	Holt 2013	Clonal group originating from genetic sweep 4
3.7.29.1.2	VN2.MSM2	MSM Clade 2	Bardsley 2020	MSM-linked strains, emerging from sweep 2
3.7.29.1.2.1	VN2.MSM2.Aus	- (part of BAPS3)	This study Ingle 2019	Australian MSM-linked, emerging from sweep 2
3.7.29.1.3	VN2.Hue	Hue2	Holt 2013	Hue subclone 2, emerging from sweep 3
3.7.29.1.4	VN2.KH1	KH1	Holt 2013	Kanh Hoa subclone 1, emerging from sweep 2
3.7.29.1.4.1	VN2.KH1.Aus	- (part of BAPS3)	This study Ingle 2019	Australian isolates, emerging from KH1
3.7.30	Middle East III	Middle East III	Holt 2012	Associated with Middle East
3.7.30.4	Israel III	Israel III	This study	Associated with Israel
3.7.30.4.1	OJC	OJC-associated	Baker 2016	Associated with the Orthodox Jewish communities in Israel, UK, USA and Europe

To facilitate communication about genotypes of epidemiological interest, we also assigned them human readable aliases (e.g., 3.6.1.1 = CipR, 3.6.1.1.1 = CipR.SEA, 3.6.1.1.2 = CipR.MSM5, see Table 2). As far as possible these aliases map to names given in previous publications, e.g., the MSM clade numbers designated in^16,17^. Most of the epidemiological groups of interest belong to Lineage 3, and detailed phylogenies for these groups are provided in Supplementary Fig. 2. In addition to being monophyletic on the tree, these higher-level genotypes were supported by FastBAPS analysis (Supplementary Fig. 1). Pairwise distances within and between genotypes of all levels are shown in Fig. 2b.

### Development and validation of SNV-based scheme for assigning genotypes

We identified marker SNVs unique to each genotype (147 SNVs in total, see Supplementary Data 2) and implemented code to assign new genomes to genotypes based on presence of these markers (see “Methods”). To validate this approach, we downloaded and genotyped 2015 additional *S. sonnei* genomes from GenomeTrakr (referred to as validation set, see Supplementary Data 1). These genomes originate from public health laboratories in three countries (*n* = 609 USA, *n* = 1325 UK, *n* = 11 Israel, *n* = 70 country unknown), with isolation dates between 2015 and 2019.

We identified 17 different genotypes, all belonging to clades 3.6 or 3.7 (Supplementary Table 2). The vast majority (70%, *n* = 1403) belonged to clade 3.6. Genotype 3.6.1.1.2 (CipR.MSM5) was the most prevalent, assigned to 26.6% of the genomes, followed by 3.6.1.1 (CipR, 19.6%) (Supplementary Table 2). The UK GenomeTrakr genomes yielded the greatest number of genotypes (*n* = 16), followed by the USA (*n* = 13); likely due to a high number of travel-associated cases. All GenomeTrakr genomes deposited from Israel were identified as 3.7.30.4 (Israel III, 9%) or 3.7.30.4.1 (OJC, 91%); these genotypes were also detected amongst UK and USA genomes.

To verify the genotyping scheme accurately captured the population structure present in the GenomeTrakr isolates, we constructed a core-genome phylogeny including both the validation set and Lineage 3 discovery set (total *n* = 3696 genomes, see “Methods”) and mapped the genotype assignments to this tree (see Supplementary Fig. 3 and Microreact https://microreact.org/project/g8BvA2JCXWaZNDyPyjsWXF). All groups of isolates sharing a genotype assignment based on marker SNVs constituted monophyletic clades within the core-genome phylogeny, consistent with the expected behaviour of the scheme. This was true for all levels in the hierarchical scheme, including clades, subclades, and higher-resolution epidemiological groups.

### Distribution of antimicrobial resistance determinants amongst *S. sonnei* genotypes

**Fig. 3:**
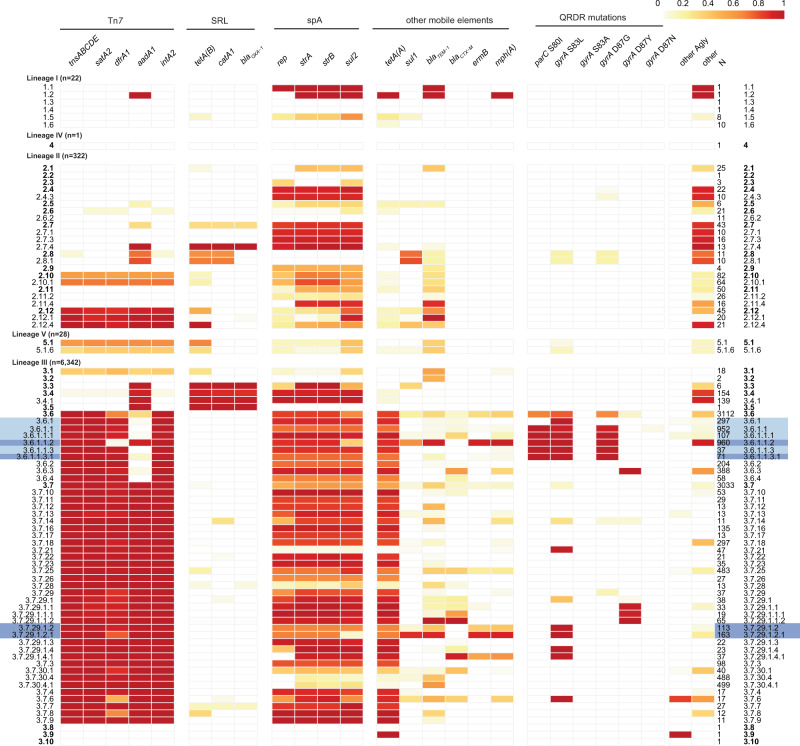
Frequencies of AMR genetic determinants within individual *S. sonnei* genotypes, calculated across 6715 genomes. Cells indicate absence (white) or presence (coloured by proportion as per legend) of each AMR determinant (columns) within each clade or higher-resolution genotype (rows). All clades are included as rows (bold labels); subclades and higher-resolution genotypes represented by ≥10 genomes are also included as distinct rows; number of genomes in each row are noted in column “N”. Light blue shading indicates fluoroquinolone resistant genotypes; dark blue shading indicates MSM-associated genotypes. Columns are grouped by typical location of the AMR determinant (labelled horizontal bars at the top): transposon Tn*7*, represented by marker genes *tnsABCDE* and class II integron In*2* integrase gene *intA2*; *Shigella* resistance locus (SRL); spA plasmid, represented by marker gene *rep*; other mobile elements; mutations in quinolone resistance determining region (QRDR). Column “other Agly” indicates proportion of genomes carrying at ≥1 additional aminoglycoside resistance gene beyond those with their own columns; column ‘other’ indicates proportion of genomes carrying ≥1 other AMR gene that is not otherwise listed (full AMR gene content per strain is available in Supplementary Data  1).

We used the genotyping scheme to facilitate exploration of the distribution of AMR determinants in the global *S. sonnei* population, by assessing the frequency of AMR genes and QRDR SNVs across genotypes (Fig. 3). For this analysis we included *n* = 6715 genomes: *n* = 1935 discovery set, *n* = 2015 validation set and a further *n* = 2765 public genomes (accessions listed in Supplementary Data 1). Most AMR determinants were associated with specific genotypes, present amongst either all or no members of each genotype (Supplementary Fig. 4).

Genes conferring resistance to first-line drugs were found in all lineages. Those associated with the spA plasmid (*sul2, tetA(A)*) were found in all lineages but were most widely distributed across clades of Lineage 3 (found in all clades) followed by Lineage 2 (81% of clades) (Fig. 3, Supplementary Fig. 4). Tn*7* transposon genes (*tnsABCDE*), the class II integron integrase (*intA2*) and AMR genes in the integron cassette (*satA2, dfrA1, aadA1*) were absent from Lineage 1 but found in Lineages 2 and 3 (40–41% of clades) and the single Lineage 5 clade (Fig. 3). This combination of markers for the chromosomally integrated MDR transposon Tn*7* was most common in clades 3.6 (99%), 3.7 (99%) and 2.12 (88%), where it was typically accompanied by spA genes (*sul2, tetA(A)*) resulting in resistance to co-trimoxazole (Fig. 3). First-line AMR genes *aadA1, tetA(B)*, *catA1* and *bla*_OXA-1_, which are known to mobilise together on the SRL, co-occurred in clades 3.3, 3.4 and 3.5, consistent with prior reports of SRL in clade Latin America IIIa (3.4)^10^. Acquired genes *bla*_TEM-1_ and *sul1* were also found at low frequencies across diverse genotypes (Supplementary Fig. 4), suggesting occasional acquisition via mobile elements.

QRDR SNVs were detected in 50.4% of all genomes, distributed across six clades and 12 subclades (Fig. 3), consistent with frequent emergence of these mutations under selection from drug exposure. Most common was GyrA-S83L (42% of genomes, six clades, 12 subclades) followed by GyrA-D87G (30.8% of genomes, five clades, ten subclades). Single mutants were most common (18.7% of genomes, five clades, 6 subclades) but double mutants were also observed in 3.6.1 (*n* = 61/6715 genomes had GyrA-S83L+GyrA-D87G). QRDR triple mutants, associated with ciprofloxacin resistance, were detected only in the CipR sublineage (genotype 3.6.1.1) which harbours GyrA-S83L+GyrA-D87G+ParC-S80I. The emergence and evolutionary dynamics of this CipR sublineage from within the Central Asia IIIa clade (genotype 3.6) were recently described in a detailed phylodynamic study of fluoroquinolone resistant isolates from diverse sources by The et al.^14^. That study divided the Central Asia III clade into two populations: Pop1, with either GyrA-D87Y or GyrA-S83L arising on two independent occasions in South Asia in the mid-1990 s; and Pop2, which arose from Pop1 genomes carrying GyrA-S83L in South Asia in the early 2000s, and then acquired GyrA-D87G and ParC-S80I to become fluoroquinolone resistant before spreading geographically^14^. Applying our new genotyping scheme to the genomes from The et al.^14^ (Supplementary Fig. 5), we confirm that Pop1 maps to clade 3.6 (*n* = 18) and its subclades 3.6.1, 3.6.2, 3.6.3, 3.6.4; and Pop2 maps to sublineage 3.6.1.1 (CipR, *n* = 239) including its subgroups 3.6.1.1.1 (CipR.SEA, *n* = 30), 3.6.1.1.2 (CipR.MSM5, *n* = 3), 3.6.1.1.3 (*n* = 16) and 3.6.1.1.3.1 (CipR.MSM1, *n* = 19).

Determinants of resistance to azithromycin and extended-spectrum cephalosporins were rare and concentrated mainly in clades 3.6 and 3.7. The plasmid-borne azithromycin resistance genes *mph*(A) and *ermB* were detected at high frequency in genotypes 3.6.1.1.2 (CipR.MSM5, *n* = 915, 95%) and 3.7.29.1.2.1 (VN2.MSM2.Aus, *n* = 147, 90%); *mph*(A) was present alone in the single 1.2.1 genome (Vietnam, 2007), and alone or with *ermB* at lower frequency (<65%) amongst other Lineage 3 genotypes (Fig. 3). Notably, *n* = 947 3.6.1.1 (CipR) genomes carried *mph*(A) in addition to Tn*7* and spA genes, rendering them resistant to azithromycin, ciprofloxacin and first-line drugs (*n* = 58, 3.6.1; *n* = 16, 3.6.1.1; *n* = 868, 3.6.1.1.2; *n* = 2, 3.6.1.1.3; *n* = 3, 3.6.1.1.3.1), leaving extended-spectrum cephalosporins as the last remaining oral drug. ESBL genes were detected only sporadically, at low frequencies in clades 3.6 (13%) and 3.7 (12%), across 25 distinct genotypes (frequency range, 0.6–100%, median 17.2%) (see Fig. 3). Carbapenemase genes were extremely rare, present in only two genomes (*bla*_OXA-66_ and *bla*_OXA-181_). Concerningly, we detected 40 genomes with resistance determinants for azithromycin, third-generation cephalosporins and fluoroquinolones, all within CipR genotype 3.6.1.1 (*n* = 16, 3.6.1.1; *n* = 11, 3.6.1.1.1; *n* = 15, 3.6.1.1.2). These genomes were isolated between 2014 and 2019, and were found in genomes from England (*n* = 21), Australia (*n* = 13), the USA (*n* = 3), Vietnam (*n* = 3) and the Netherlands (*n* = 2) (further details below).

### Application to public health surveillance data from Australia, England and USA

To demonstrate how the *S. sonnei* genotyping framework can facilitate the rapid tracking and reporting of emerging AMR trends across jurisdictions, we applied it to genomic surveillance data from Victoria, Australia (*n* = 644), England (*n* = 2867) and USA (*n* = 711) generated over a 4-year period (2016–2019) (data in Supplementary Data 1). The data represents all cultured isolates submitted to the Microbiological Diagnostic Unit Public Health Laboratory in Australia (42% of all *S. sonnei* notifications in Victoria)^4^ and all those sent to the Public Health England Gastrointestinal Bacteria Reference Unit^28^ (provided direct from the reference laboratories for the present study); and ~5% of those notified in the USA (sourced from the public GenomeTrakr database^33^). The total time taken to generate genotyping reports (including QRDR mutations) for all *n* = 4222 isolates was ~40 sec – 1 min per isolate using Mykrobe, with raw Illumina sequence files (fastq format) as input.

**Fig. 4:**
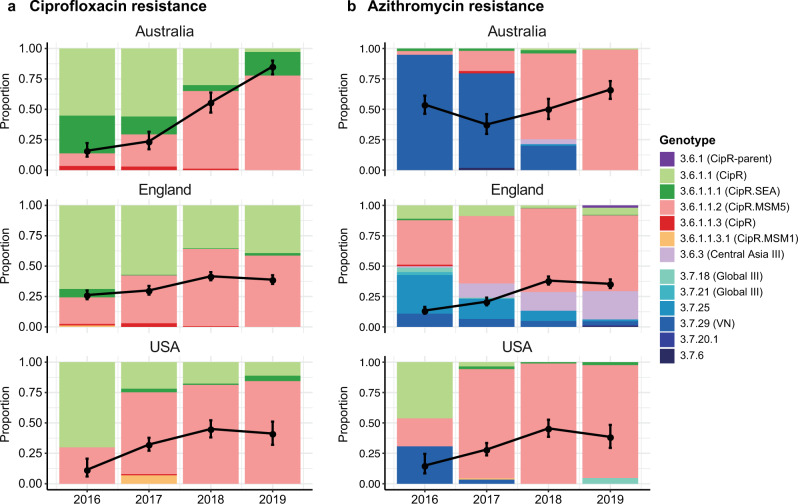
Prevalence and genotype breakdown of ciprofloxacin and azithromycin resistant *S. sonnei* in three surveillance regions (Australia, England and USA) from 2016 to 2019. In each plot, black lines indicate the proportion of genomes that are predicted resistant to (**a**) ciprofloxacin (defined as presence of ≥3 QRDR mutations) or (**b**) azithromycin (defined as carrying *mph*(A)). Error bars indicate 95% confidence intervals for the proportion resistant (*n* = 144–182 for Australia, *n* = 610–869 for England, *n* = 87–313 for USA). Stacked bars indicate the relative abundance of each genotype among resistant isolates, coloured by genotype as per legend.

Figure 4a shows the annual frequency of fluoroquinolone resistance (defined as presence of 3 QRDR mutations) in each country, and the distribution of genotypes amongst the resistant isolates. Increasing fluoroquinolone resistance rates are evident amongst the *S. sonnei* samples from each country, beginning at ≤25% in 2016 and reaching 85% in Australia, 39% in England and 44% in USA in 2019 (black lines, Fig. 4a). All fluroquinolone resistant genomes belonged to genotypes within the CipR sublineage (3.6.1.1), with the subgroup 3.6.1.1.2 (CipR.MSM5) accounting for a steadily increasing proportion of resistant isolates in each country, from ≤30% in 2016 to 78% in Australia, 59% in England and 85% in USA in 2019 (pink bars, Fig. 4a). These results are consistent with local epidemiological outbreaks of *S. sonnei* in MSM communities in England^16,17^ and Australia^4,26^. Notably however, the common nomenclature makes it easy to identify several epidemiologically important patterns: (i) all resistant isolates in all three countries derive from the previously-described CipR sublineage 3.6.1.1 that emerged from South Asia in the early 2000s; (ii) the reported spread of resistant *S. sonnei* in MSM communities in Australia and England involves the same strain (this was not clear from previous reports, as the strain was named “MSM clade 5” in the English studies and formed a subgroup within the “BAPS3 cluster” in the Australian studies); (iii) this strain represents a clonal subgroup of the CipR sublineage (genotype 3.6.1.1.2, CipR.MSM5) that has disseminated intercontinentally over the last few years and become responsible for the majority of fluoroquinolone resistant *S. sonnei* infections in all three countries. (Note an additional two English isolates of genotype 3.6.1.1 from 2019 carried *gyrA*-S83L and *parC*-S80I plus the *qnrS* gene, which likely combine to confer fluoroquinolone resistance^34^).

**Fig. 5:**
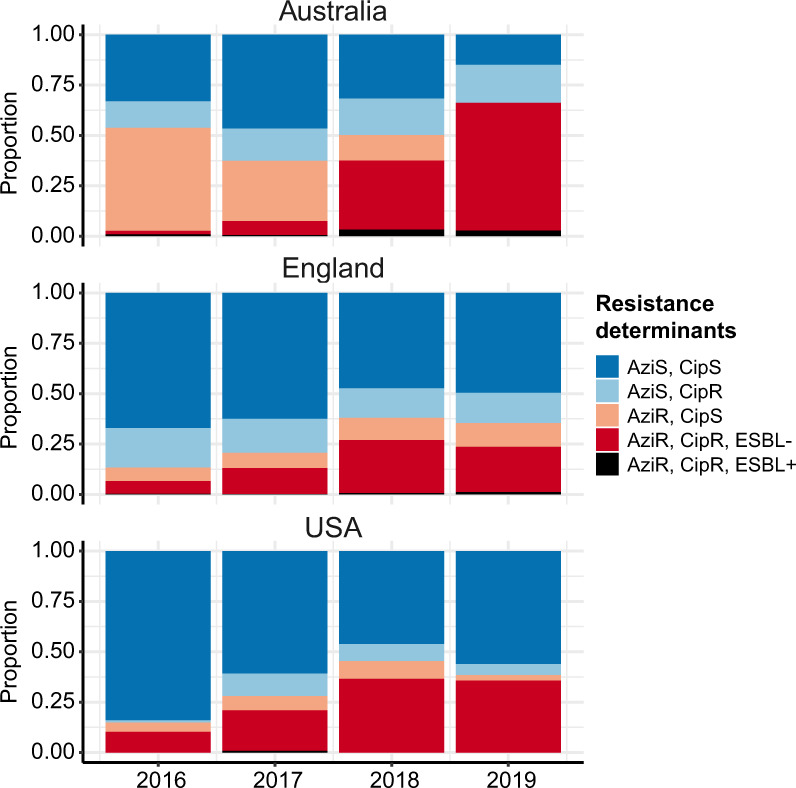
Prevalence of combined resistance to ciprofloxacin and azithromycin amongst genomes from each surveillance region. Stacked bar colours indicate the relative abundance of different combinations of resistances, predicted from genomes: CipR, ciprofloxacin resistant (defined as presence of ≥3 QRDR mutations); AziR, azithromycin resistant (defined as carrying *mph(A)*); ESBL+, presence of extended-spectrum beta-lactamase (ESBL) gene associated with resistance to third generation cephalosporins (*bla*_CTX-M-14_, *bla*_CTX-M-15_, *bla*_CTX-M-27_*, bla*_CTX-M-55_*, bla*_CTX-M-134_).

Figure 4b shows the annual frequency of azithromycin resistance (predicted by presence of *mph*(A)) in each country, and the genotypes responsible. Resistance rates were high (>50%) in Australia across the whole period, reflecting documented outbreaks in the MSM community^4^. In England and the USA, rates increased between 2016 and 2019, from 13 to 38% in England and 15 to 45% in the USA (black lines, Fig. 4b). The genotype distributions amongst *mph*(A) + genomes differed markedly between countries in 2016, dominated by 3.7.29 (VNclone, 95%) in Australia, 3.7.25 (MSM4, 32%) and 3.6.1.1.2 (CipR.MSM5, 36%) in England, and 3.6.1.1 (CipR, 46%), 3.7.29 (VNclone, 31%) and 3.6.1.1.2 (CipR.MSM5, 23%) in the USA (see barplot, Fig. 4b). Notably though, the contribution of 3.6.1.1.2 (CipR.MSM5) increased dramatically in each country, and in 2019 accounted for 99% of *mph*(A) + isolates in Australia, 62% in England and 93% in USA (bars, Fig. 4b). Thus in 2019, the majority of predicted azithromycin resistant isolates in all three countries were also predicted to be resistant to ciprofloxacin and first line drugs. Concerningly, the proportion of total *S. sonnei* genomes with combined resistance determinants for azithromycin, ciprofloxacin and first-line drugs was high (36–66%) in all three countries in 2019 (Fig. 5).

Whilst ESBL genes were rare across the *S. sonnei* surveillance data (7.8% in Australia, 17.5% in England, 1% in USA, see Supplementary Data 1), concerningly 201 genomes carried ESBL genes in addition to *mph*(A), and 36 of these were QRDR triple mutants belonging to 3.6.1.1 (CipR). The latter were concentrated in genotypes 3.6.1.1, 3.6.1.1.1 and 3.6.1.1.2 and comprised 13 Australian genomes (2% of total, 2016–2019, all three genotypes), 20 English genomes (0.7% of total, in 2016 and 2018–2019, all three genotypes) and 3 USA genomes (0.4% of total, 2017, only 3.6.1.1 and 3.6.1.1.1) (Fig. 5). Consistent with the overall picture of ESBL genes in *S. sonnei* described above (Fig. 3), there was no evidence of strong linkage between specific ESBL genes and particular genotype backgrounds in the public health surveillance data for this period (Supplementary Data 1). This was true even amongst CipR/AziR/ESBL + genomes, which included eight unique combinations of genotype and *bla*_CTX-M_ allele (3–6 per country, Supplementary Fig. 6), consistent with multiple independent acquisitions of different AMR plasmids in different settings contributing to the march towards pan-resistance to oral drugs.

## Discussion

Here, we provide a global framework for *S. sonnei*, and identify marker SNVs that can be used to easily position newly sequenced isolates into this framework, without the need for time-consuming comparative genomics or phylogenetic analysis. We demonstrate that the population structure of *S. sonnei* can be represented by a robust maximum likelihood phylogeny and define within it 137 subtrees on the basis of pairwise divergence and epidemiological coherence, which we designate as hierarchically nested genotypes. Furthermore, we provide a software package implemented within the Mykrobe^32^ code base, which can identify both the *S. sonnei* genotype and QRDR SNVs direct from short-read sequence files in a few seconds.

The genotyping scheme was constructed with a view towards stability, prioritising as markers SNVs that are found in highly conserved core genes that are not under adaptive selection in the population. We also endeavoured to make the scheme backwards compatible by identifying and designating unique genotypes for *S. sonnei* genetic clusters defined in previous studies on the basis of epidemiological features (see Table 2). We further aimed to ensure genotypes are interpretable, with stable numerical identifiers that convey relationships between genotypes, and human readable aliases that convey relevant epidemiological information where appropriate (see Table 2). The genotyping scheme can be readily expanded in future, by adding new genotypes and corresponding SNV markers as new clusters of agreed epidemiological importance are identified based on phylogenetic analyses. We envisage managing future updates via an international working group consisting of epidemiological, public health and genomics experts who utilise the scheme, similar to the approach proposed for a recent *Neisseria gonorrhoeae* typing scheme^35^, and have adopted clear versioning to facilitate compatibility between laboratories and over time. Importantly, our approach provides genotype definitions and nomenclature that are stable and transparent, not dependent on comparative analysis or on any specific sequencing assay or software package, although we provide an implementation in the Mykrobe v0.9.0 software for convenience. The Mykrobe implementation was tested on all Illumina reads used in this study, and matching long (ONT) reads for eight of these genomes. Correct genotypes were returned in all cases (providing the --ont flag to Mykrobe when using the long reads as input). This overall approach is designed to facilitate easy communication between laboratories and jurisdictions, and straightforward comparison of pathogen populations over time, allowing for rapid identification of clonal dissemination and AMR trends.

To demonstrate the utility of the genotyping scheme for public health applications, we applied it to summarise large genomic surveillance datasets from three jurisdictions in Australia, England and the USA. The results provide a straightforward view of temporal trends in the populations of *S. sonnei* causing disease in each jurisdiction. They also clearly identify common ciprofloxacin and azithromycin resistant clones that have spread globally and are now present in all three jurisdictions, which was previously not obvious from individual studies, as there was no common nomenclature.

The genotyping approach introduced here could greatly simplify the bioinformatics procedures required for routine genomic surveillance of *S. sonnei* in reference laboratories, the first step of which usually involves comparison of newly sequenced genomes to those from prior cases in the same jurisdiction (to monitor local trends) and/or other jurisdictions (to monitor introduction of new strains and patterns of regional spread). Notably, the public health labs in Australia, England and USA from which we sourced genomic surveillance data (Fig. 4) all utilise genome-wide SNV-based phylogenetics for *S. sonnei* analysis^17,26^.

Reliance on whole genome comparisons and phylogenetic inference is considerably slower than genotyping and requires expertise and background knowledge both to conduct the analysis and to interpret the results. In contrast, using the genotyping framework, raw sequence data can be turned into simple informative identifiers for each strain without reference to other genomes or databases (and without needing to assemble the genome). The resulting genotyping information can be easily interpreted, compared and stored in (non-sequence) databases for future reference, facilitating epidemiological investigations without need for direct comparisons with any other genome sequences. Detailed phylogenetic analysis can then be applied to subsets of isolates that share the same or similar genotypes, if needed to address specific questions (e.g., relating to emerging local outbreaks or transmission networks). Notably, such phylogenetic analyses are essential to identify the emergence of novel clusters from within currently defined genotypes. If a high-quality *S. sonnei* genome sequence lacked all the lineage-specific SNV markers in the genotyping scheme, this would suggest it belongs to a novel lineage. Such cases could be investigated further using phylogenomics, and the scheme expanded to include the novel lineage. While this is expected to be rare, it may well occur as more sequencing is undertaken in Africa and other regions currently under-represented by the available genomic data.

For example, scientists investigating azithromycin-resistant *S. sonnei* isolated from MSM in Switzerland recently reported identifying the strains as belonging to the same clones spreading through MSM communities in England. To achieve this identification, they had to download English genome data, compare their newly isolated genomes to these using read mapping, and construct and interpret whole genome phylogenies^29^. A similar study using WGS to investigate a ciprofloxacin-resistant outbreak in California used the same informatics approach to conclude that the local strain belonged to the previously described ciprofloxacin resistant lineage originating in South Asia^23^. Another recent study from Switzerland used WGS to investigate an increase in ESBL *S. sonnei* using a combination of cgMLST and phylogenetics^36^. Using our new genotyping approach, all of these identifications could be made within minutes of obtaining sequence data, with no need for external comparative data, background knowledge of *S. sonnei* genetics, or complex computational infrastructure and expertise (see Supplementary Note 1, Supplementary Fig. 7, Supplementary Table 3 and Supplementary Data 3). The rapid identification of genomically-related isolates via genotyping could be used to facilitate timely public health responses to shigellosis outbreaks.

A potential alternative to SNV-based genotyping is cgMLST, available for *Shigella/E. coli* via EnteroBase^9^. The scheme consists of 2513 genes as markers (~half the genome), including loci under positive selection such as *gyrA* and *parC*, which are used to define core genome sequence types (cgSTs). EnteroBase also assigns cgSTs to hierarchical clusters (HC) with stable names, using the HierCC algorithm^9^, at various locus-distance thresholds (2, 5, 10, 20, etc). HC5 is widely used for detecting outbreak clusters of *Salmonella* and *E. coli* in foodborne surveillance^37–41^, however clustering of *S. sonnei* at HC5 (or even HC10) breaks apart epidemiologically recognised outbreak clusters such as the MSM clades 3.6.1.1.3.1 (CipR.MSM1), 3.6.1.1.2 (CipR.MSM5) and 3.7.25 (MSM4) (see Supplementary Fig. 8), making them hard to track. We speculate that the over-division of *S. sonnei* by cgMLST may be due to the fact that many alleles are affected by deletion or insertion sequence activity, as is common in *S. sonnei*^42^. Notably our focused genotyping scheme is designed to utilise a small number of highly stable loci, avoiding this issue. In addition, cgST numbers and HierCC cluster numbers are arbitrarily assigned and carry no meaning beyond being unique identifiers, whereas hierarchical naming systems such as those used in our scheme (and those adopted for *M. tuberculosis*, *S*. Typhi and SARS-CoV-2^43^ schemes) are much more informative as they communicate relationships between clusters. Perhaps for these reasons, there is to-date only one published report of *S. sonnei* analysis that utilised cgMLST for public health investigation^36^, and they too relied on additional SNV-based phylogenetics to place their local isolates in the context of global populations as this was not easily ascertained using cgMLST (see Example 2 in Supplementary Note 1, Supplementary Data 3).

In conclusion, while genomics is increasingly becoming a standard tool for surveillance of *S. sonnei* and other pathogens in public health reference laboratories, this poses computational and epidemiological challenges in terms of analysis, interpretation and communication of genome-derived data across discipline and jurisdictional boundaries. The genotyping framework and universal nomenclature for *S. sonnei* established here provides a solution for many of these issues, and provides a structure to enable clear communication between public health and basic science research groups. Importantly, it will facilitate monitoring of the emergence and spread of AMR *S. sonnei* clones, at local and global levels, which will become increasingly important as public health agencies face the emerging threat of pan-resistant *S. sonnei*.

## Methods

### Single nucleotide variants (SNVs) and phylogenetic analysis – discovery dataset

The discovery dataset consisted of 1935 high quality previously published *S. sonnei* genomes, sequenced using Illumina platforms. Source information for all genomes (year of collection, geographic origin, etc) was extracted from their respective publications^4,7,10–13,15,16^ (Supplementary Table 1 and  Supplementary Data 1). Genomes were mapped to the *S. sonnei* reference genome 53 G (accession NC_016822) using RedDog (v1beta11; https://github.com/katholt/RedDog). Briefly, RedDog maps reads with Bowtie2 v2.2.9^44^ with the sensitive local parameter, then uses SAMtools v1.1^45^ to retain high quality SNV calls (phred score ≥20, read depth >5, removes heterozygous calls). SNVs detected in repetitive regions where SNV calls are dubious (e.g. insertion sequences, phage) were removed (see Figshare, 10.26180/5f1a443b19b2f). SNVs associated with recombination were identified and excluded using Gubbins v2.3.2^46^ (*n* = 208 SNVs across 10 genomes). The final alignment consisted of 23,673 SNVs across 1935 genomes. Ancestral alleles at these SNV sites were extracted from five *E. coli* genomes (accessions CP019005, CP031916, CP019961, CP034399 and CP019259) using the mapping procedure described above, and were included in the alignment for the purpose of outgroup rooting the tree. A maximum likelihood (ML) phylogeny was inferred using IQ-TREE v2^47^ using a GTR substitution model (Fig. 1b). An interactive form of the tree is available in Microreact, at https://microreact.org/project/fG2N7huk9oZNCaVHu8rukr.

### Defining clades and subclades of the genotyping scheme

The discovery set was analysed to define appropriate SNV thresholds for assigning genomes to genotypes based on pairwise genetic distances. Pairwise SNV distances were calculated for all pairs of, and thresholds were selected by examining the distribution of pairwise SNV distances (Fig. 2a), to define clusters at three levels: lineage (600 SNVs), clade (215 SNVs) and subclade (100 SNVs). To cluster discovery set genomes at these thresholds, we applied hierarchical clustering (using *hclust* function in R, with complete linkage) to the pairwise SNV distance matrix. The resulting dendrogram was then cut at the aforementioned thresholds (using the R function *cutree*) to cluster isolates into discrete groups representing lineages, clades and subclades. The resulting groups were compared to the ML phylogeny to check that each was monophyletic (using the function *is.monophyletic* in the *ape* package^48^ for R); a small number of groups were non-monophyletic and were broken up into smaller groups (2 groups at clade level, *n* = 388 isolates; 3 groups at subclade level, *n* = 52 isolates) to result in a final set of monophyletic clusters. The SNV alignment was also analysed using the R package *fastbaps*^49^ to partition the data into groups using Bayesian clustering (using the *hc* method for prior optimisation, and two levels of clustering using *multi_res_baps*) (Supplementary Fig. 1a). Pairwise SNV distances within and between our final genotype groups (Fig. 2b), and within and between FastBAPS clusters for comparison (Supplementary Fig. 1b), were calculated from the SNV alignments using the *dna.dist* function in the *ape* package^48^. Isolates belonging to previously defined epidemiological groups were located in the ML tree, and used to identify subtrees corresponding to each set of group members. For epidemiological groups defined on the basis of AMR determinants, the presence of these determinants (identified using SRST2^50^ and the QRDR SNVs) was used to determine the boundaries of the local subtree sharing the defining features of the group (Table 2).

### Developing and implementing SNV-based genotyping scheme

We identified potential marker SNVs for each of the 147 genotypes by mapping SNVs onto the branches of the ML phylogeny using SNPPar^51^ with default parameters. A total of 980 homoplasic SNVs were identified and excluded from consideration as markers. For most genotypes multiple markers mapped to the defining branch; we prioritised synonymous changes within core genes (*n* = 138) over nonsynonymous (*n* = 8) or intergenic SNVs (*n* = 1), and where multiple synonymous SNVs were available we prioritised genes with the lowest ratio of nonsynonymous:synonymous SNVs (calculated from SNPPar output) as these are less likely to be under selection in the population and thus serve as stable marker SNVs. The list of SNV markers is given in Supplementary Data 2.

We modified the Mykrobe genotyping software^32^ to probe for these marker SNVs and assign *S. sonnei* genotypes. Probes to detect changes at specific *S. sonnei* QRDR codons (GyrA-83, GyrA-87, ParC-80) were also included in the *S. sonnei* panel in Mykrobe^32^ (v0.9.0) software available at https://github.com/Mykrobe-tools/mykrobe, using the probe panel stored in Figshare (10.6084/m9.figshare.13072646). Mykrobe outputs were then parsed using a custom Python script which tabulates results across a set of samples and summarises the support for each genotype call (https://github.com/katholt/sonneityping). We tested the Mykrobe *S. sonnei* genotyper using the discovery genome set as input (Illumina reads, fastq format), to confirm that the genotype and QRDR mutations reported by this implementation were correct. Code was also tested using Oxford Nanopore (ONT) long reads (fastq format) using the --ont flag in Mykrobe.

### Validating the genotyping scheme on independent data

We used an independent validation dataset (i.e., genomes not included in the discovery set used to define the scheme) a total of 2015 *S. sonnei* genomes downloaded from the GenomeTrakr project^33^ on 7 May 7 2019 (listed in Supplementary Data 1). All genomes had been sequenced using Illumina platforms. The validation and discovery datasets were subjected to mapping, SNV calling and phylogenetic analysis as described above, generating a recombination-filtered core-genome alignment of 32,138 SNVs in 3696 isolates (*n* = 260 SNVs identified as recombinant across 11 genomes), and a ML phylogeny (Supplementary Fig. 3, interactive tree available in Microreact at https://microreact.org/project/g8BvA2JCXWaZNDyPyjsWXF). All genomes were assigned a genotype using Mykrobe^32^ v0.9.0, and these were compared to the ML phylogeny to check that each genotype was monophyletic as expected (using the function *is.monophyletic* in the *ape* package^48^ for R). In addition, we confirmed that all marker SNVs remained non-homoplasic amongst this larger set of genomes, using output from SNPPar (a total of 2003 homoplastic SNVs were identified amongst this expanded set of genomes).

### Analysis of AMR determinants amongst genotypes

Genotypes were identified as above, and AMR determinants were identified using SRST2^50^ and the CARD database^52^ for a further 2644 genomes sourced from public databases, and results combined with those from the discovery and validation sets yielding a total of *n* = 6595 *S. sonnei* genomes for analysis. Genotypes with at least ten representatives in this data set (total *n* = 57 genotypes), and AMR genes detected in at least two genomes, were included in the analysis of AMR frequencies within genotypes (Fig. 3 and Supplementary Fig. 4). Data were analysed in R and visualised using the *pheatmap* and *ggridges* packages.

To assess the correspondence between genotypes defined here within the CipR clade (genotype 3.6.1.1 and its subtypes) and the two subpopulations (Pop1, Pop2) defined previously by The et al.^14^, we constructed a ML phylogeny including all genomes analysed in The et al.^14^ and at least one representative from each Lineage 3 clade and each genotype in clade 3.6. Genomes which had not already been included in the discovery set were genotyped, and subjected to mapping and SNV calling for inclusion in phylogenetic analysis as described above, generating an alignment of 9178 SNVs and an ML phylogeny (Supplementary Fig. 5, interactive tree available in Microreact at https://microreact.org/project/kMRoFFXxkB6JAn9bgBAdMz).

### Application to public health surveillance data

We applied the *S. sonnei* genotyping framework to analyse 4222 genomes sequenced by public health reference labs between 2016 and 2019 in Australia (*n* = 644^4^), England (*n* = 2867^16,17^), and the USA (*n* = 711, from GenomeTrakr^33^ as of 7 May 2019). All genomes were genotyped, and AMR determinants were identified as above. Azithromycin resistance was predicted based on presence of *mph*(A), ESBL/carbapenemase production based on known beta-lactamase alleles (CTX-M, OXA-66, OXA-181), and ciprofloxacin resistance based on the combination of three QRDR mutations (GyrA-S83L, GyrA-D87Y and ParC-S80I). Data were analysed in R and visualised using the *ggplot2* package (Figs. 4–5).

### Reporting summary

Further information on research design is available in the Nature Research Reporting Summary linked to this article.

## Supplementary information

Supplementary Information

Descriptions of Additional Supplementary Files

Supplementary Data 1

Supplementary Data 2

Supplementary Data 3

Reporting Summary

## Data Availability

Supplementary Data 1 lists all genome data used, with read accessions, source information, genotype calls and AMR determinants Marker SNVs used to define genotypes are tabulated in Supplementary Data 2 and GitHub, 10.5281/zenodo.4609813; and in Mykrobe panel format in Figshare, 10.6084/m9.figshare.13072646 AMR genes were detected from CARD database v3.0.8, available at https://card.mcmaster.ca/download Regions of the *S. sonnei* 53 G reference genome excluded from SNV calling are available in Figshare, 10.26180/5f1a443b19b2f Interactive annotated trees are available in Microreact for the following collections: Discovery data: https://microreact.org/project/fG2N7huk9oZNCaVHu8rukr Validation data: https://microreact.org/project/g8BvA2JCXWaZNDyPyjsWXF CipR clade: https://microreact.org/project/kMRoFFXxkB6JAn9bgBAdMz

## Code availability

Mykrobe v0.9.0 is available for download at https://github.com/Mykrobe-tools/mykrobe

Instructions for running Mykrobe v0.9.0 for *S. sonnei* and tabulating the results is available at https://github.com/katholt/sonneityping (10.5281/zenodo.4609813)
