# Supplementary material for: Global population structure and genotyping framework for genomic surveillance of the major dysentery pathogen, Shigella sonnei
Source: Nat Commun. 2021 May 11;12:2684. doi: 10.1038/s41467-021-22700-4 (PMC8113504; doi:10.1038/s41467-021-22700-4)
Supplement: Supplementary file 1 — Supplementary Information [file 41467_2021_22700_MOESM1_ESM.pdf]

Global population structure and genotyping framework for genomic surveillance of the major dysentery pathogen, *Shigella sonnei*

Jane Hawkey, Kalani Paranagama, Kate S. Baker, Rebecca J. Bengtsson, François-Xavier Weill, Nicholas R. Thomson, Stephen Baker, Louise Cerdeira, Zamin Iqbal, Martin Hunt, Danielle J. Ingle, Timothy J. Dallman, Claire Jenkins, Deborah A. Williamson, Kathryn E. Holt

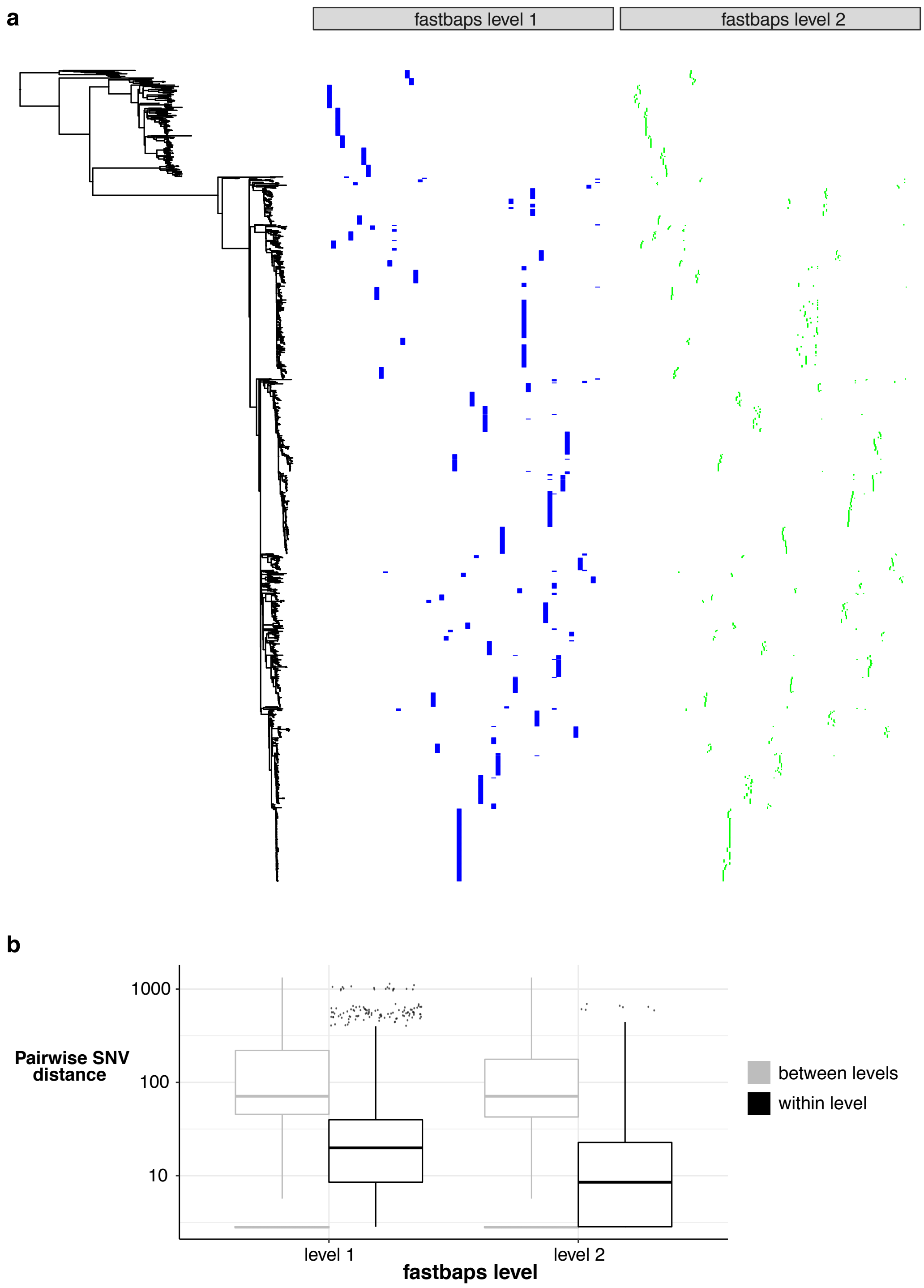

Supplementary Figure 1: Partitions detected in the discovery genomes using fastbaps (Bayesian hierarchical clustering on SNV matrix). a fastbaps partitions (levels 1, blue; and 2, green) plotted against the maximum likelihood phylogeny. **b** Boxplots of pairwise SNV distances (log scale, N=1,873,081 pairwise distances) between discovery genomes at the two levels of fastbaps partitioning. Boxes indicate the median (bold line), 25th to 75th percentiles (box), and the 5th and 95th percentile (whiskers), with outliers shown as points. Note that this form of clustering results in two levels that while hierarchical (level 2 nested within level 1) and generally monophyletic on the tree, are not coherent in terms of genetic divergence (approximated by SNV distance), which is a desirable property of a genotyping scheme. Boxes indicate

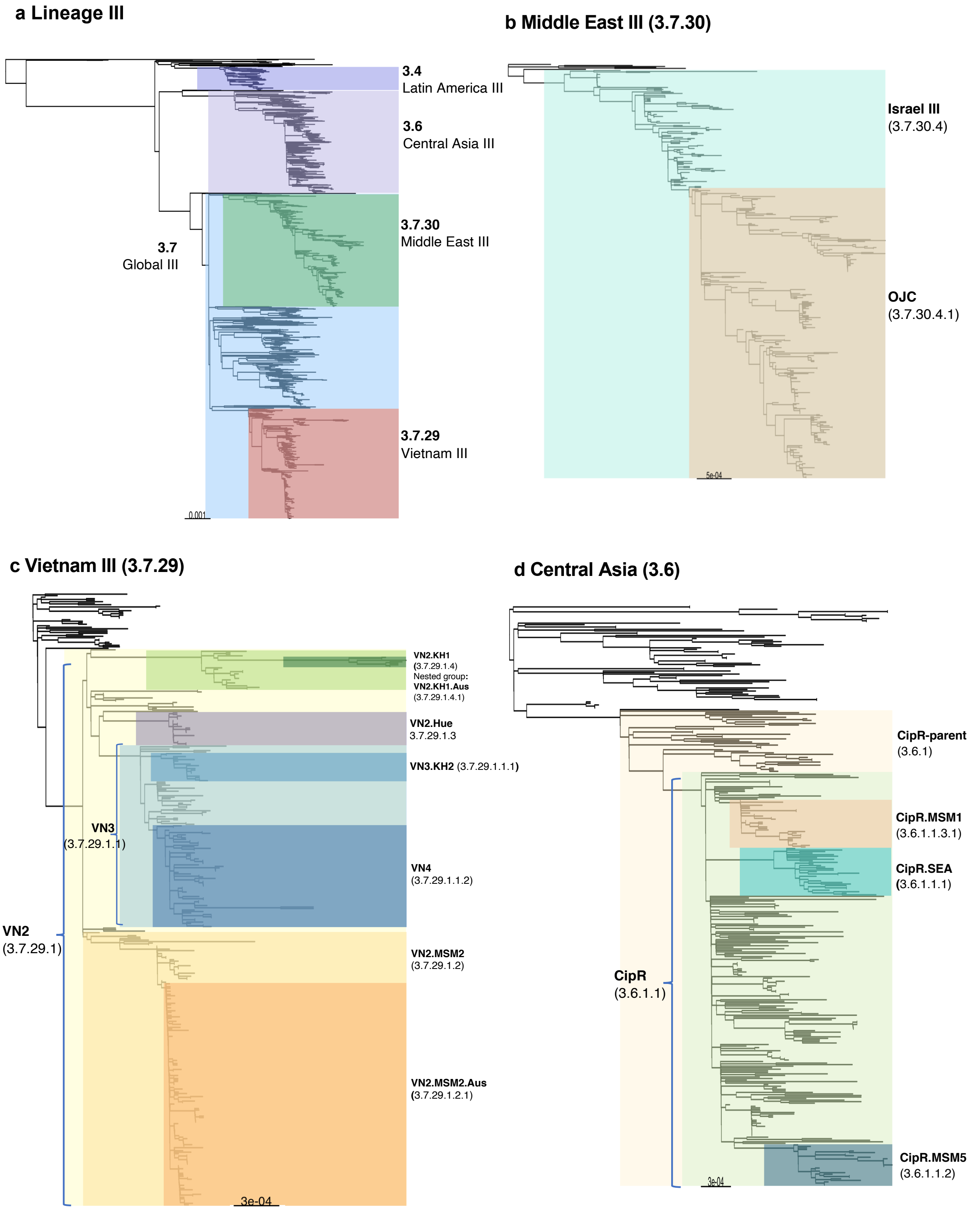

Supplementary Figure 2: Maximum likelihood phylogenies for sublineages of epidemiological interest within Lineage 3 (discovery set genomes).

a ML phylogeny of all discovery genomes in Lineage 3, with major clades/subclades highlighted. **b-d** Subtrees of the Lineage 3 ML phylogeny showing detail within the three most common clades/subclades; major epidemiological groups described in prior publications (Table 2) are highlighted and labelled. These can also be explored within the interactive version of the annotated discovery set phylogeny, available in Microreact (https://microreact.org/project/fG2N7huk9oZNCaVHu8rukr).

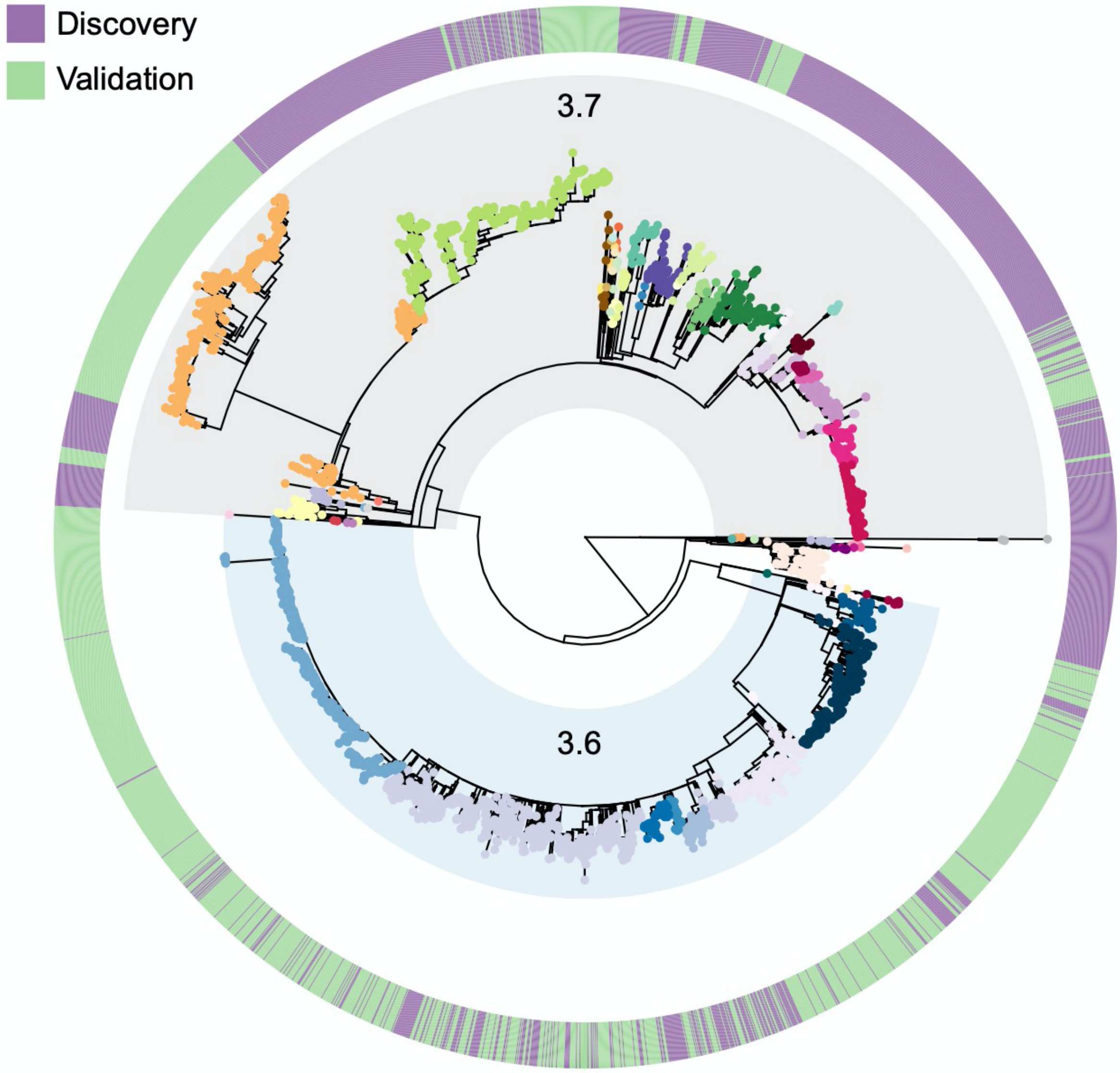

Supplementary Figure 3: Maximum likelihood phylogeny of Lineage 3, including discovery set genomes + 2,016 validation genomes from GenomeTrakr. Tips are coloured by genotype, with the major clades (3.6 and 3.7) highlighted and labelled (each set of tips assigned to the same genotype was confirmed to be monophyletic in the tree, see Methods). Outer ring indicates membership of discovery vs validation sets (as per legend), showing intermingling of genomes from both sources. Interactive version of this tree and annotations are available in Microreact (https://microreact.org/project/g8BvA2JCXWaZNDyPyjsWXF).

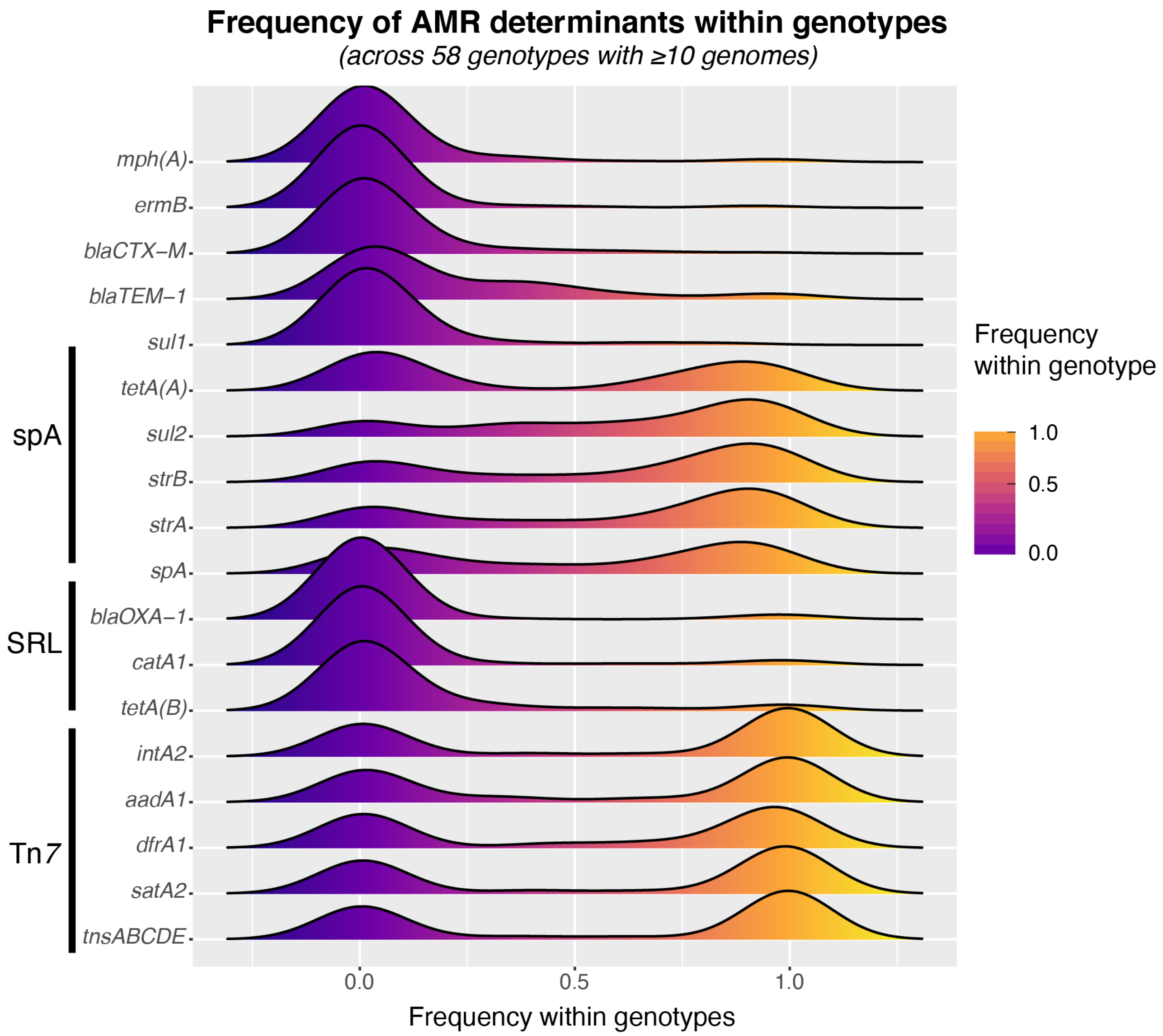

Supplementary Figure 4: Distribution of frequencies of AMR determinants within common *S. sonnei* genotypes. Each row indicates the frequency distribution, amongst 58 genotypes with ≥10 genomes sampled, for an acquired AMR gene or associated mobile element (transposon Tn*7* genes *tnsABCDE*, class II integron In*2* integrase gene *intA2*, spA plasmid replication gene *rep*; AMR genes typically associated with each of these are indicated with labelled bars on the left). Most genes are observed at frequencies of ~0 (absent from all genomes) or ~1.0 (present in all genomes) within individual genotypes. Notably the spA plasmid-associated genes are present at high frequency in most genotypes. Gene frequencies within specific individual genotypes are shown in Figure 3.

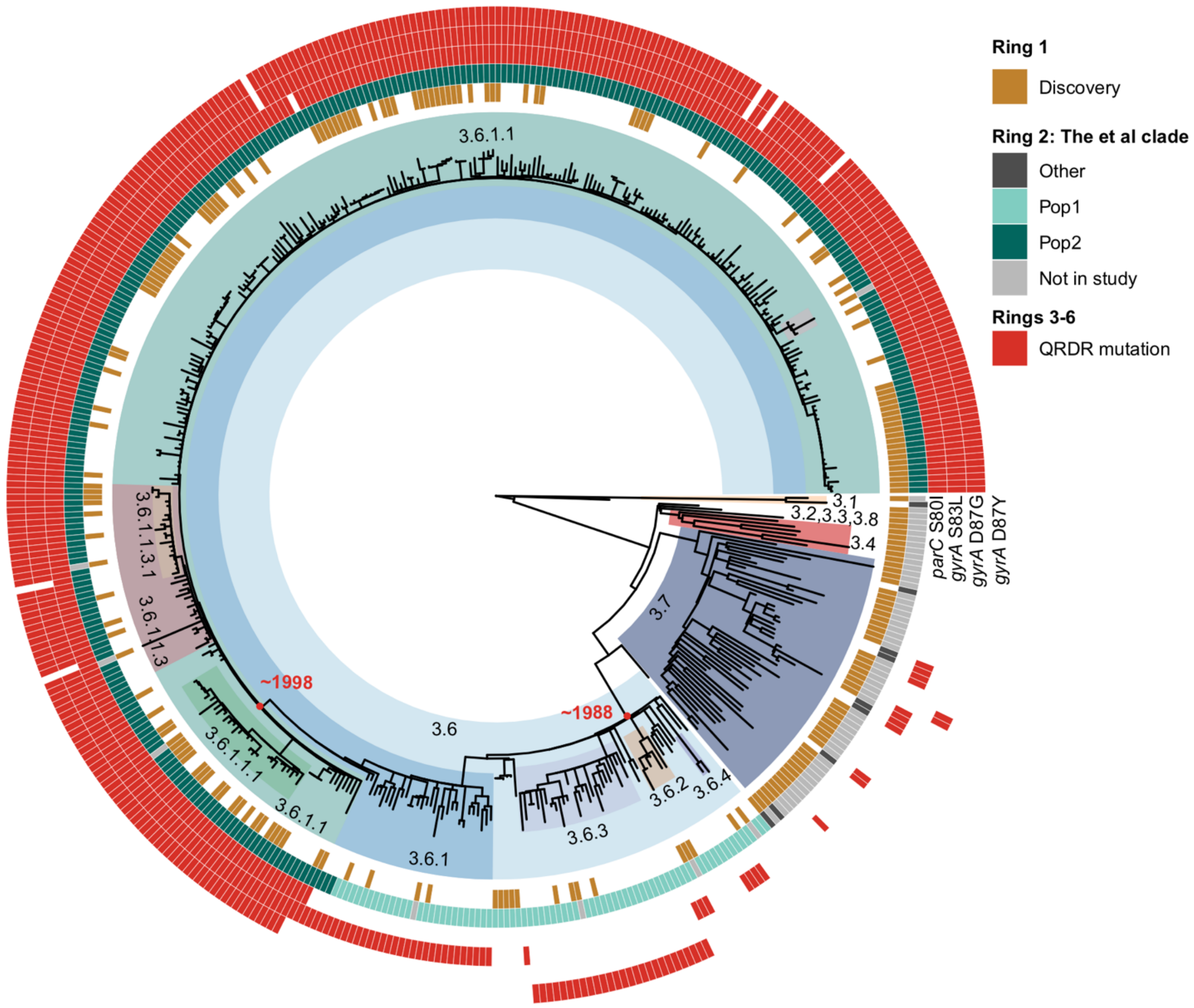

Supplementary Figure 5: Maximum likelihood phylogeny for all Lineage III genomes from The *et al*, 2019. Genotypes are highlighted and labelled. Ring one of heatmap denotes whether the strain is a representative from the discovery dataset, ring two indicates which population from The et al each genome belongs to (as per legend), and rings three-six indicate the presence (red) or absence (white) of the four QRDR mutations (rings are labelled with gene and mutation). Red dots on nodes and text indicate approximate date of divergence of these clades as per The et al, 2019. Interactive version of this tree and annotations are available in Microreact (https://microreact.org/project/kMRoFFXxkB6JAn9bgBAdMz).

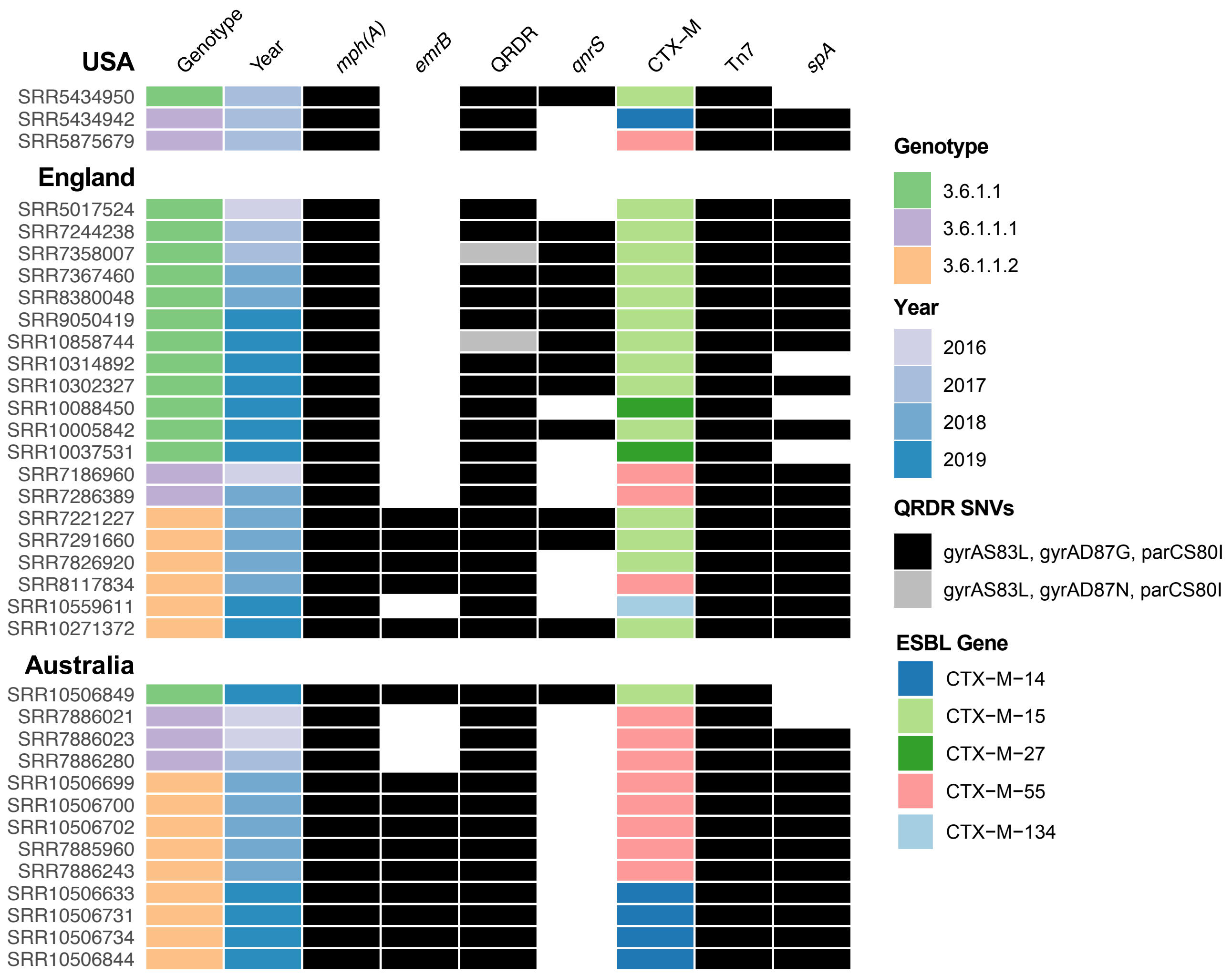

Supplementary Figure 6: Features of *S. sonnei* genomes carrying resistance determinants for all three of azithromycin, ciprofloxacin and third generation cephalosporins. Heatmap cells are coloured as per legend.

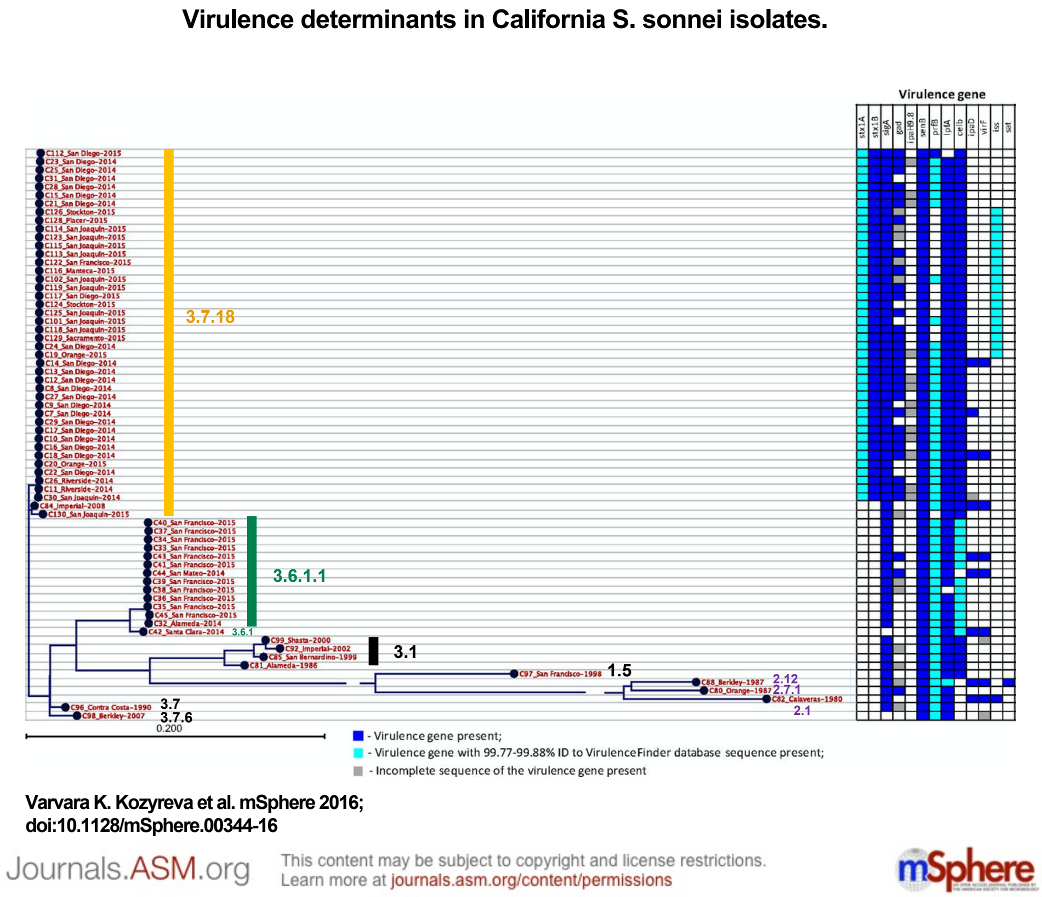

Supplementary Figure 7: ***S. sonnei*** **phylogeny from Kozyreva et al, 2016,** ***mSphere*** **1(6):e00344-16; reproduced with genotypes annotated.**

This study used WGS to investigate isolates from two outbreaks in California in the context of local historical isolates: an outbreak of ciprofloxacin resistant (Cip^{R}) cases in San Francisco, and an unrelated outbreak of Shiga toxin-positive cases in San Diego and San Joaquin. Figure shown is reproduced from Figure 3 of Kozyreva et al, reproduced under Creative Commons Attribution 4.0 International license, onto which we have annotated genotypes as determine by Mykrobe analysis of Illumina reads. Inferences made from genotyping replicated key findings inferred from the published genome-wide SNP calling and phylogenetic anlaysis, which required combined analysis with 188 global isolates (see Supplementary Notes).

Original figure legend: "Virulence determinants in California *S. sonnei* isolates. Virulence determinants: *stx1A*, Shiga toxin 1, subunit A, variant a; *stx1B*, Shiga toxin 1, subunit B, variant a; *sigA*, Shigella IgA-like protease homolog; *gad*, glutamate decarboxylase; *ipaH9.8*, invasion plasmid antigen; *senB*, plasmid-encoded enterotoxin; *prfB*, P-related fimbriae regulatory gene; *lpfA*, long polar fimbriae; *celb*, endonuclease colicin E2; *ipaD*, invasion protein *S. flexneri*; *virF*, VirF transcriptional activator; *iss*, increased serum survival; *sat*, secreted autotransporter toxin."

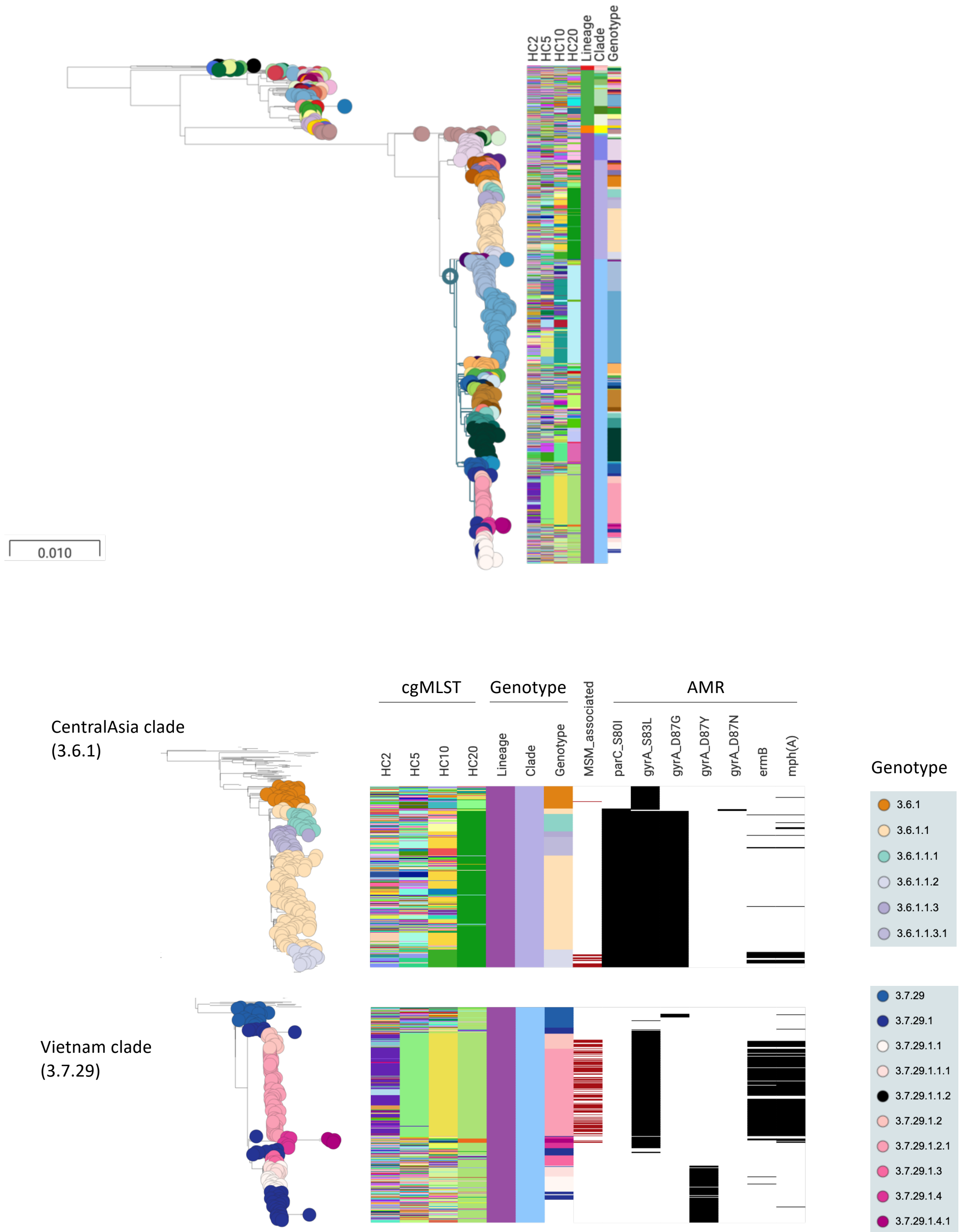

Supplementary Figure 8. cgMLST clustering is not phylogenetically informative in *S. sonnei*. Whole genome SNV trees for **a** *S. sonnei*, and **b** the dominant subclades 3.6.1 and 3.7.29, annotated with results of hierarchical clustering of cgSTs using the Enterobase cgMLST scheme for *E. coli/Shigella*. Tips are coloured by genotype (as per legend); columns 1-4 indicate HC2, HC5, HC10, HC20 clustering of cgMLST profiles exported from Enterobase, columns 5-7 indicate lineage, clade and genotype under our scheme. Interactive version available at :https://microreact.org/project/pYymuNfwCMdEMnGz64ni2i

Supplementary Table 1: Genomic *S. sonnei* studies forming the discovery dataset

Study	Year published	No of isolates	Description
Holt *et al*[1]	2012	132	Collection of globally representative *S. sonnei* isolates
Holt *et al*[2]	2013	263	*S. sonnei* isolated from Vietnam over 15-year period
Baker *et al*[3]	2017	323	*S. sonnei* from nine Latin America and Caribbean countries
Baker *et al*[4]	2018	312	*S. sonnei* from England and France, includes MSM strains
The *et al*[5]	2015	93	Ciprofloxacin resistant *S. sonnei* from Bhutan
The *et al*[6]	2016	66	Ciprofloxacin resistant *S. sonnei* from South Asia
Baker *et al*[7]	2016	437	*S. sonnei* from Orthodox Jewish communities in Israel, Europe, US & Canada
Ingle *et al*[8]	2019	364	*S. sonnei* from Victoria, Australia. Includes MSM strains

Supplementary Table 2: Summary of genotypes detected in the GenomeTrakr validation dataset

Genotype	Name	Number (%)	Years of isolation	Countries (% of genotype)
**3.6.1**	CipR-parent	143 (7.1%)	2016-2019	UK (58%)<br>USA (36%)<br>Unknown (6%)
**3.6.1.1**	CipR	396 (19.6%)	2015-2019	UK (83%)<br>USA (15.4%)<br>Unknown (1.7%)
**3.6.1.1.1**	CipR.SEA	19 (0.9%)	2016-2019	UK (74%)<br>USA (26%)
**3.6.1.1.2**	CipR.MSM5	535 (26.6%)	2016-2019	UK (64%)<br>USA (34%)<br>Unknown (2%)
**3.6.1.1.3**	CipR	9 (0.4%)	2016-2018	UK (89%)<br>USA (11%)
**3.6.1.1.3.1**	CipR.MSM1	26 (1.3%)	2015-2017	UK (65%)<br>USA (35%)
**3.6.2**	Central Asia III	42 (2.1%)	2016, 2018	UK (100%)
**3.6.3**	Central Asia III	231 (11.3%)	2016-2019	UK (99%)<br>USA (1%)
**3.7.16**	Global III	26 (1.3%)	2016, 2018	UK (100%)
**3.7.18**	Global III	1 (0.04%)	2017	USA (100%)
**3.7.21**	Global III	37 (1.8%)	2016-2019	UK (95%)<br>USA (5%)
**3.7.29.1**	VN2	2 (0.1%)	2018	UK (100%)
**3.7.29.1.2**	VN2.MSM	66 (3.3%)	2016-2018	UK (86%)<br>USA (14%)
**3.7.29.1.2.1**	VN2.MSM.Aus	11 (0.5%)	2016-2018	UK (55%)<br>USA (45%)
**3.7.30.1**	Middle East III	18 (0.9%)	2016-2019	UK (100%)
**3.7.30.4**	Israel III	334 (16.6%)	2015-2019	Israel (0.3%)<br>UK (4%)<br>USA (83%)<br>Unknown (13%)
**3.7.30.4.1**	OJC	119 (5.9%)	2016-2019	Israel (8%)<br>UK (91%)<br>USA (1%)

Supplementary Table 3: Genotyping of data from study of *S. sonnei* detected in the MSM community in Switzerland.

The study by Vladimira et al 2018 (*Swiss Medical Weekly*, doi:10.4414/smw.2018.14645) describes three cases of *S. sonnei* in the Swiss MSM community, whose origins were investigated by comparing their genomes to those of sporadic local cases and recently sequenced MSM-associated cases from the UK. Table shows the links between local MSM isolates and UK isolates concluded from the published genome-wide read mapping, SNP calling and phylogenetic analysis; alongside genotypes as determined by Mykrobe analysis of Illumina reads, which replicate key findings (see Supplementary Notes).

Accession	Study ID	Designation in published study	Genotype
ERR2220854	case3	12 SNVs from UK MSM cluster 1	3.7.25 (MSM4)
ERR2220855	case2	Clustered; 16 SNVs from UK MSM cluster 7	3.6.1.1.2 (CipR.MSM5)
ERR2220856	case1		3.6.1.1.2 (CipR.MSM5)
ERR2220857	outgroup1	-	3.7.25 (MSM4)
ERR2220858	outgroup2	-	3.7.25 (MSM4)
ERR2220859	outgroup3	-	3.7
ERR2220860	outgroup4	-	3.7.21
ERR2220861	outgroup5	-	3.7.18
ERR2220862	outgroup6	-	3.7.15
ERR2220863	outgroup7	-	3.7.3

Supplementary Note 1

Example 1 – Investigation of two Californian *S. sonnei* outbreaks
Kozyreva et al, 2016, *mSphere* 1(6):e00344-16

This study used WGS to investigate isolates from two outbreaks in California in the context of local historical isolates: an outbreak of ciprofloxacin resistant (Cip^R) cases in San Francisco, and an unrelated outbreak of Shiga toxin-positive cases in San Diego and San Joaquin. Genome-wide mapping-based SNV calling and phylogenetic analysis showed the two outbreaks formed tight clusters that were distant from one another, and from local historical isolates, in the phylogenetic tree. They also constructed additional genome-wide phylogenies incorporating 188 isolates from prior studies of globally distributed *S. sonnei*[1], in order to identify which of the main lineages (I, II, III, Global III, IV) their outbreak clusters belonged to; this identified the San Diego/San Joaquin as lineage III, and the San Francisco Cip^R outbreak as belonging to Global III and clustering with the South-Asia clade of isolates sharing the same three QRDR mutations explaining the Cip^R phenotype.

We downloaded the Illumina reads for the California isolates via the accessions indicated and genotyped them using Mykrobe (compute time, 1 minute per genome). Supplementary Figure 7 reproduces Figure 3 from Kozyreva *et al*, onto which we have annoted the Mykrobe-derived genotypes. This identified the historical isolates as lineage 1 (n=1), lineage 2 (n=3) or clades within lineage 3 (genotypes 3.1 and 3.7, n=6), distinct from the two outbreaks and consistent with the whole-genome phylogeny. Genomes from the San Francisco Cip^R outbreak were identified as 3.6.1.1, the well-described CipR clade, and carrying its typical 3 QRDR mutations; those from the San Diego/San Joaquin outbreak were identified as genotype 3.7.18, a subclade of Global III (3.7). Genotype 3.7.18 was defined on the basis of isolates from Latin America, consistent with epidemiological investigations of the outbreak which implicated travel from Mexico as the likely route of introduction of the outbreak strain into California. Thus findings from genotyping replicate those derived by the authors using more complex comparative methods.

Notably, Kozyreva *et al* found that all genomes from their outbreak carried the Shiga-toxin virulence genes *stx1A* and *stx1B*. We screened all 3.7.18 genomes in our dataset (n=297) but were unable to detect these genes in our genomes, suggesting that the presence of these virulence factors was limited to this outbreak, and is not a widespread feature of the genotype.

Example 2 – Study of ESBL *S. sonnei* in Switzerland
Campos-Madueno *et al* 2020 (*Antimicrobial Agents and Chemotherapy,* doi:10.1128/AAC.01057-20)

This study examines 25 *S. sonnei* genomes isolated between 2016 and 2019 in Switzerland; 14 were resistant to third-generation cephalosporins and 11 were sensitive. The authors sequenced the genomes of these isolates and subjected them to cgMLST using Enterobase[9], which identified 18 cgSTs. They then constructed a tree comprising their novel genomes, the global collection of *S. sonnei* from Holt *et al* 2012[1] and other isolates from Enterobase with matching cgSTs by extracting SNVs from the cgMLST loci. Based on the SNV tree the authors identified four monophyletic clusters associated with different ESBL genes, three (clusters 1-3) grouping with Global III and one (cluster 4) grouping with Lineage III but outside Global III. Notably two of these clusters comprised multiple cgSTs; i.e. cgST alone was not enough to identify the clusters. The authors concluded that

there were multiple plasmids carrying ESBL genes present in different clusters, with no single plasmid responsible for ESBL strains in Switzerland. They also identified a small number of isolates with matching cgSTs in the Enterobase database, noting that (i) all Swiss ESBL cgSTs identified in 2019 were also detected in the same year in UK and France; and (ii) cluster 1 shared a cgST with two older isolates from Iran and Egypt.

We downloaded the assemblies for the Swiss genomes using the accessions provided in the study, simulated reads, and genotyped them using Mykrobe (compute time, 30 seconds per genome). The results are indicated in the 'Genotype' and 'Genotype Name' columns in Supplementary Data 3, and identified five genotypes amongst ESBL-positive strains, which map to the four clusters identified by cgST plus an additional singleton strain (3.6.1.1). Genotyping yielded essentially the same information as the combined cgMLST and tree analysis, while also providing clearer links to known MSM clusters circulating in Europe and identifying cluster 4 and the singleton strain as ciprofloxacin resistant: (i) cluster 4 belongs to genotype 3.6.1.1.2 (CipR.MSM5); (ii) clusters 2 and 3 both belong to another UK MSM-associated genotype within Global III, 3.7.25 (MSM4); (iii) cluster 1 belongs to genotype 3.7.30.1, which originated in the Middle East (Middle East III).

While both cgSTs and genotypes provide a straightforward way of searching databases for related strains, this example demonstrates that cgMLST can over-classify genomes in ways that obscure close relationships, as genomes belonging to the same genotype frequently belong to multiple cgSTs that were not readily identified as related without specific further analysis (by inspecting pairwise SNV distances, locus-variant distances or hierarchical clustering). In contrast, the hierarchical nomenclature used in our genotyping scheme ensures that defining genotypes at higher resolution has no disadvantage, as relationships between genotypes can easily be recognised without need for comparative analysis.

Example 3 – Study of *S. sonnei* detected in the MSM community in Switzerland
Vladimira et al 2018 (*Swiss Medical Weekly*, doi:10.4414/smw.2018.14645)

This study describes three cases of *S. sonnei* in the local MSM community in Switzerland. To determine the relationships of these isolates to one another, to sporadic *S. sonnei* in Switzerland, and to recently sequenced MSM-associated isolates from the UK, the authors sequenced their three cases plus another seven non-MSM-related local *S. sonnei*, and inferred a tree incorporating these plus a selection of 32 publicly available MSM-associated sequences from the UK[10] using genome-wide read mapping, SNV calling and neighbour-joining tree construction. Based on the tree, the authors concluded that: (i) the MSM cases were not closely related to the local non-MSM strains; (ii) two MSM cases were closely related to one another, and clustered with the previously reported UK MSM cluster 1[10]; (iii) the third case was not related to this pair of cases, but clustered with UK MSM cluster 7.

We downloaded the Illumina reads for the Swiss isolates via the accessions indicated (see Supplementary Table 3) and genotyped them using Mykrobe (compute time, 1 minute per genome). The results are indicated under 'Genotype' and 'Genotype Name' in Supplementary Table 3, and show: (i) the MSM cases had distinct genotypes from the local non-MSM cases; (ii) two cases share the same genotype 3.6.1.1.2 which matches a known ciprofloxacin-resistant UK MSM strain (CipR.MSM5); (iii) the third case is of unrelated genotype 3.7.25 which matches another known UK MSM strain (CipR.MSM4). Hence the same key conclusions could be drawn from the simple and fast genotype analysis of the novel Swiss strains alone, as could be drawn

from the more complex comparative whole-genome phylogenetics analysis incorporating additional public data from previous MSM studies.

Supplementary References

1. Holt, K. E. *et al.* Shigella sonnei genome sequencing and phylogenetic analysis indicate recent global dissemination from Europe. *Nat. Genet.* **44**, 1056–1059 (2012).
2. Holt, K. E. *et al.* Tracking the establishment of local endemic populations of an emergent enteric pathogen. *Proc. Natl. Acad. Sci. U. S. A.* **110**, 17522–17527 (2013).
3. Baker, K. S. *et al.* Whole genome sequencing of Shigella sonnei through PulseNet Latin America and Caribbean: advancing global surveillance of foodborne illnesses. *Clin. Microbiol. Infect.* **23**, 845–853 (2017).
4. Baker, K. S. *et al.* Genomic epidemiology of Shigella in the United Kingdom shows transmission of pathogen sublineages and determinants of antimicrobial resistance. *Sci. Rep.* **8**, 7389 (2018).
5. The, H. C. *et al.* Introduction and establishment of fluoroquinolone-resistant Shigella sonnei into Bhutan. *Microb. Genomics* **1**, (2015).
6. The, H. C. *et al.* South Asia as a Reservoir for the Global Spread of Ciprofloxacin-Resistant Shigella sonnei: A Cross-Sectional Study. *PLoS Med.* **13**, e1002055 (2016).
7. Baker, K. S. *et al.* Travel- and community-based transmission of multidrug-resistant Shigella sonnei lineage among international Orthodox Jewish communities. *Emerg. Infect. Dis.* **22**, 1545–1553 (2016).
8. Ingle, D. J. *et al.* Co-circulation of Multidrug-resistant Shigella Among Men Who Have Sex With Men in Australia. *Clin. Infect. Dis.* **69**, 1535–1544 (2019).
9. Zhou, Z., Alikhan, N. F., Mohamed, K., Fan, Y. & Achtman, M. The EnteroBase user's guide, with case studies on Salmonella transmissions, Yersinia pestis phylogeny, and Escherichia core genomic diversity. *Genome Res.* (2020) doi:10.1101/gr.251678.119.
10. Dallman, T. J. *et al.* Use of whole-genome sequencing for the public health surveillance of Shigella sonnei in England and wales, 2015. *Journal of Medical Microbiology* (2016) doi:10.1099/jmm.0.000296.
