# Supplementary material for: Global population structure and genotyping framework for genomic surveillance of the major dysentery pathogen, Shigella sonnei
Source: Nat Commun. 2021 May 11;12:2684. doi: 10.1038/s41467-021-22700-4 (PMC8113504; doi:10.1038/s41467-021-22700-4)
Supplement: Supplementary file 2 — Descriptions of Additional Supplementary Files [file 41467_2021_22700_MOESM2_ESM.pdf]

Descriptions of Additional Supplementary Files

Supplementary Data 1
Description: Metadata for all S. sonnei genomes used in this study.

Supplementary Data 2
Description: Details for marker SNVs used for the genotyping scheme.

Supplementary Data 3
Description: Genotyping data from a study of ESBL S. sonnei in Switzerland. The study by Campos-Madueno et al 2020 (Antimicrobial Agents and Chemotherapy, doi:10.1128/AAC.01057-20) examines 25 S. sonnei genomes isolated in Switzerland between 2016 and 2019, of which 14 were resistant to third-generation cephalosporins. Table gives the accessions for the corresponding genome assemblies; ESBL genes reported in each; summary of conclusions regarding clustering and lineage assignments drawn by the authors based on cgMLST and SNV tree analysis; and genotypes inferred from Mykrobe analysis of the genome assemblies, which replicate key findings (see Supplementary Text).
