# Supplementary material for: Global population structure and genotyping framework for genomic surveillance of the major dysentery pathogen, Shigella sonnei
Source: Nat Commun. 2021 May 11;12:2684. doi: 10.1038/s41467-021-22700-4 (PMC8113504; doi:10.1038/s41467-021-22700-4)
Supplement: Supplementary file 6 — Reporting Summary [file 41467_2021_22700_MOESM6_ESM.pdf]

nature research

Corresponding author(s): Jane Hawkey

Last updated by author(s): Mar 17, 2021

Reporting Summary

Nature Research wishes to improve the reproducibility of the work that we publish. This form provides structure for consistency and transparency in reporting. For further information on Nature Research policies, see our Editorial Policies and the Editorial Policy Checklist.

Statistics

For all statistical analyses, confirm that the following items are present in the figure legend, table legend, main text, or Methods section.

n/a	Confirmed	
[ ]	[x]	The exact sample size (n) for each experimental group/condition, given as a discrete number and unit of measurement
[x]	[ ]	A statement on whether measurements were taken from distinct samples or whether the same sample was measured repeatedly
[x]	[ ]	The statistical test(s) used AND whether they are one- or two-sided<br>*Only common tests should be described solely by name; describe more complex techniques in the Methods section.*
[x]	[ ]	A description of all covariates tested
[x]	[ ]	A description of any assumptions or corrections, such as tests of normality and adjustment for multiple comparisons
[x]	[ ]	A full description of the statistical parameters including central tendency (e.g. means) or other basic estimates (e.g. regression coefficient) AND variation (e.g. standard deviation) or associated estimates of uncertainty (e.g. confidence intervals)
[x]	[ ]	For null hypothesis testing, the test statistic (e.g. F, t, r) with confidence intervals, effect sizes, degrees of freedom and P value noted<br>*Give P values as exact values whenever suitable.*
[x]	[ ]	For Bayesian analysis, information on the choice of priors and Markov chain Monte Carlo settings
[x]	[ ]	For hierarchical and complex designs, identification of the appropriate level for tests and full reporting of outcomes
[x]	[ ]	Estimates of effect sizes (e.g. Cohen's d, Pearson's r), indicating how they were calculated

Our web collection on statistics for biologists contains articles on many of the points above.

Software and code

Policy information about availability of computer code

Data collection	N/A
Data analysis	Read mapping to the reference genome was performed using RedDog v1b11, which uses Bowtie2 v2.2.9 and SAMtools v1.1<br>Recombination was identified and removed using Gubbins v2.3.2<br>Phylogenetic inference was performed with IQ-TREE v2<br>Assignment of SNVs to branches of the phylogeny was done using SNPPar v1<br>Antimicrobial resistance genes were detected from sequence reads using SRST2 v0.2.0<br>Genotyping was performed with Mykrobe v0.9.0, and parsing of Mykrobe output was performed using a custom parser that can be found at https://github.com/katholt/sonneityping (v20210201)<br>Visualisation was performed in R v3.6.3, using packages ape v5.3, fastbaps v1, pheatmap v1.0.12, ggridges v0.5.2

For manuscripts utilizing custom algorithms or software that are central to the research but not yet described in published literature, software must be made available to editors and reviewers. We strongly encourage code deposition in a community repository (e.g. GitHub). See the Nature Research guidelines for submitting code & software for further information.

Data

Policy information about availability of data

All manuscripts must include a data availability statement. This statement should provide the following information, where applicable:

- Accession codes, unique identifiers, or web links for publicly available datasets
- A list of figures that have associated raw data
- A description of any restrictions on data availability

Supplementary Data 1 lists all genome data used, with read accessions and source information
Supplementary Data 2 lists marker SNVs used to define genotypes
Regions of the S. sonnei 53G reference genome excluded from SNV calling are available in Figshare (doi: 10.26180/5f1a443b19b2f)
Interactive annotated trees (in microreact) are available for (i) Discovery data: https://microreact.org/project/fG2N7huk9oZNCaVHu8rukr ; (ii) Validation data: https://microreact.org/project/g8BvA2JCXWaZNDyPyjsWXF ; (iii) CipR clade: https://microreact.org/project/kMRoFFXxkB6JAn9bgBAdMz
Instructions for running Mykrobe v0.9.0 and parsing the output for S. sonnei is available at https://github.com/katholt/sonneityping
Mykrobe S. sonnei probe panel is available in Figshare https://doi.org/10.6084/m9.figshare.13072646

Field-specific reporting

Please select the one below that is the best fit for your research. If you are not sure, read the appropriate sections before making your selection.

☐ Life sciences ☐ Behavioural & social sciences ☒ Ecological, evolutionary & environmental sciences

For a reference copy of the document with all sections, see nature.com/documents/nr-reporting-summary-flat.pdf

Ecological, evolutionary & environmental sciences study design

All studies must disclose on these points even when the disclosure is negative.

Study description	The goal of this study was to produce a genotyping framework for S. sonnei, and to then use this framework to assess trends in the evolution and spread of important S. sonnei genotypes.
Research sample	Our sample consists of 6,715 publicly available S. sonnei genomes that have been sequenced on the Illumina platform. Full details (including source, published study, Bioproject ID and accession) of all genomes included can be found in Supplementary Data 1. Our rationale for our collection was to include all publicly available genome data, and to make our collection as globally representative as possible, but were limited by what genome data is currently available.
Sampling strategy	Our sampling strategy was to obtain all publicly available short read genome data that met standard QC thresholds (described in Data exclusions)
Data collection	Data was downloaded from the SRA or ENA by JH and KP using Monash University's high performance computing system, with all accessions stored in a single spreadsheet, including BioProject ID and published study describing the data. For data included in published studies, metadata (including geographic origin, year of isolation, etc) was extracted from the supplementary information of those studies and included in our master spreadsheet.
Timing and spatial scale	Discovery (n=1,935) and validation (n=2,015) data used to create and validate the genotyping scheme was collated during the first half of 2019. The remaining 2,765 genomes were collated during the second half of 2019 and the first half of 2020, to include as much public data as possible before manuscript submission.
Data exclusions	Genomes were excluded if they did not meeting one or more of the following mapping criteria:<br>- coverage of reference genome 53G was <90%<br>- total percentage of reads mapped to reference genome 53G was <50%<br>- average depth across the 53G reference genome was <10x<br>- the ratio of heterozygous to homozygous SNV calls was >1
Reproducibility	We validated our genotyping scheme on 2,015 genomes from GenomeTrakr (deposited on or before May 7 2019). Validation was performed by genotyping strains using Mykrobe, and we compared their called genotype with their phylogenetic placement in a tree of all discovery and validation genomes.
Randomization	N/A, not statistically comparing groups to one another
Blinding	N/A, this study was not a clinical trial

Did the study involve field work? ☐ Yes ☒ No

Reporting for specific materials, systems and methods

We require information from authors about some types of materials, experimental systems and methods used in many studies. Here, indicate whether each material, system or method listed is relevant to your study. If you are not sure if a list item applies to your research, read the appropriate section before selecting a response.

Materials & experimental systems

n/a	Involved in the study
☒	☐ Antibodies
☒	☐ Eukaryotic cell lines
☒	☐ Palaeontology and archaeology
☒	☐ Animals and other organisms
☒	☐ Human research participants
☒	☐ Clinical data
☒	☐ Dual use research of concern

Methods

n/a	Involved in the study
☒	☐ ChIP-seq
☒	☐ Flow cytometry
☒	☐ MRI-based neuroimaging
